# Understanding driving forces of food waste separation intention to enhance regional and local solid waste planning: application of PLS-SEM and multi-group analysis

**DOI:** 10.1007/s11356-024-34485-2

**Published:** 2024-08-05

**Authors:** Cuong Le Dinh, Takeshi Fujiwara, Song Toan Pham Phu, Habuer

**Affiliations:** 1 Graduate School of Environmental and Life Science, Okayama University, Okayama, 700-8530 Japan; 2 Faculty of Chemical Technology – Environment, The University of Danang – University of Technology and Education, 48 Cao Thang Street, Hai Chau District, Danang City, 550000 Vietnam; 3 Graduate School of Environmental, Life, Natural Science and Technology, Okayama University, Okayama, 700-8530 Japan

**Keywords:** Food-waste separation, Social behaviour, Partial least squares structural equation modelling (PLS-SEM), Circular economy, Vietnam

## Abstract

This study extended the theory of planned behaviour (TPB) and applied it to analyse influencing factors of food waste separation intention (FSI) among households in central Vietnam. Partial least squares structural equation modelling (PLS-SEM) was employed to analyse the data. The heterogeneity of factors contributing to FSI in cities of three levels was examined using multi-group analysis (MGA). The results indicate that attitudes, subjective norms (SN), perceived behavioural control (PBC), awareness of benefit (AB), information publicity (IP), facility availability (FA), and trust significantly affected FSI of households. MGA results indicated the heterogeneity of impacts of PBC and attitude on FSI of households among three municipal levels. The results will serve as basic data for waste officers, solid-waste management project leaders, non-governmental organisations (NGOs), and other related stakeholders to lay the foundation of food waste management planning in terms of regional scale and local scale. This study will also aid the creation of a circular economy by providing a scientific base for enhancing food waste separation at source in central Vietnam.

**Supplementary Information:**

The online version contains supplementary material available at 10.1007/s11356-024-34485-2.

## Introduction

Traditional linear economies compromise ecosystems and waste management approaches, causing negative environmental consequences (Riba et al. 2020). A circular economy (CE) solves problems of a traditional economy by enhancing resource exploitation and reducing environmental pollution (Kuah and Wang 2020). Recent advances in recycling technology aid the establishment of circular economies. However, advances in technology alone cannot combat all problems associated with solid-waste management (SWM) and formation of CE (Pathak et al. 2023). Human issues are critical when dealing with SWM issues and establishing a CE, especially in developing countries (Schröder et al. 2020).

Vietnam is a developing country, experiencing challenges imposed by linear economies and poor SWM (MONRE 2019). The Vietnamese government has decreed that a shift to a CE will serve as a systematic solution to the current problems and challenges posed by the linear economy and SWM (GSRV 2022; NASR 2020). With a population of approximately 100 million (GSOV 2022), Vietnam generates daily about 60,000 tons of domestic waste while 70% of domestic waste has been transferred to landfills without conducting waste separation at source (WSS) (MONRE 2019, 2021). This has caused negative impacts on the environment and society, namely landfill overloading, waste of resources, and social unrest. Recently, Vietnamese environmental law requires that domestic solid waste must be separated into food waste, recyclable waste, and other waste to head to CE (NASR 2020).

Although food waste is an important part of SWM and potential to head to CE (Das et al. 2024; Wani et al. 2024), however, the majority of food waste, accounting for the largest proportion (about 50–70%) of domestic waste generation in Vietnam, being dumped at landfills without any explicit regional planning for food waste management (MONRE 2019; Song Toan et al. 2019, 2021). Despite existence of compost facilities, the quality of output is not proper for returning back to material circularity (Bao et al. 2023). One of the major causes is low effectiveness of food waste separation at source (FSS) (Song Toan et al. 2019). Effectiveness of FSS strongly relies on household participation (Gundupalli et al. 2017; Pakpour et al. 2014) due to large proportion of household waste in municipal solid waste generation (Dangi et al. 2011; Noor et al. 2020). Thus, social studies for enhancing household FSS is gaining momentum in Vietnam—a developing country.

Psychological and behavioural studies on enhancing FSS are still limited in Vietnam and other developing countries. The majority of social studies focused on WSS and waste separation intention (WSI) in Ghana (Adu-Gyamfi et al. 2023; Tang et al. 2023), China (Cudjoe et al. 2020; Wang et al 2021; Wang et al. 2020; Wang et al. 2019), Vietnam (Nhung 2023; Phuong et al. 2015), and Pakistan (Li et al. 2023). Regarding food-waste-related studies, a study focused on food waste reduction in Malaysia (Chun T’ing et al. 2021) while there were studies about organic waste separation in Vietnam (Loan et al. 2017) and FSS in Malaysia (Ghani et al. 2013; Ng et al. 2021). Thus, this study will fill in the research gap by strongly focusing on FSS in Vietnam and enriching knowledge for enhancing FSS in developing countries.

In addition, direct measurement of FSS behaviour is arduous as that action happens in homes and people’s self-reported results are imprecise (Oehman et al. 2022; Quested et al. 2020). Moreover, household intention is considered an important precursor to actual behaviour (Ajzen 1991, 2015; Hu et al. 2021; Rhodes et al. 2014). Accordingly, a plethora of methods have been utilised as analytical frameworks to focus on scrutinising the driving forces of household intention for WSS and FSS. Theory of planned behaviour (TPB) has been demonstrated to be effective in examining behavioural intention regarding FSS (Ghani et al. 2013; Govindan et al. 2022; Ma et al. 2018; Nhung 2023). Due to the complex mechanism of human behaviour, the original TPB itself cannot fully explain the driving force towards the behaviour intention of waste generators (Tang et al. 2022; Tonglet et al. 2004). Hence, the need for expansion of original TPP has been denoted in a host of studies about FSS and food-waste separation intention (FSI).

Regarding pro-environmental studies, awareness of benefits of environmentally friendly actions is proven to be a factor contributing to human intention (Adu-Gyamfi et al. 2023; Cudjoe et al. 2020). Thus, enhancement of FSI is expected if citizens can acknowledge environmental benefits of FSS. Moreover, information explosion is ubiquitously affecting human daily life, in that, information publicity will considerably contribute to human intention (Li et al. 2023; Tang et al. 2023; Wang et al. 2019). Information publicity is also expected as an effective tool for providing information for environmentally friendly actions such as FSS (Meng et al. 2019). Thereby, information publicity is expected to have positive effect on improvement of household FSI in Vietnam. Additionally, limitation of resources such as time and knowledge would hinder FSS (Phuong et al. 2015; Wang et al. 2020). Hence, situational factors should be considered to have more thorough understanding of FSS. Unlike other types of waste, people, who conduct FSS, handle a material which has a strong odour or attracts pests (Benyam et al. 2020). Facility availability is also demonstrated to be significantly related to FSI (Ng et al. 2021). Thus, the available facilities are expected to help citizens overcome problems of FSS and enhance FSI. It was also asserted that trust has prominent role on facilitating human cooperation for pro-environmental actions (Van Lange et al. 2013). Trust also has an influence on human intention and should be examined for understanding pro-environmental behaviour such as WSI (Phuong et al. 2015). Therefore, this study expanded TPB by adding additional latent variables, namely awareness of benefit (AB), information publicity (IP), situational factor (SF), facility availability (FA), and trust to deepen understanding of factors contributing to FSI.

Moreover, regional planning is an important content within SWM (Eugene et al. 2016; Karimi et al. 2022; Rigamonti et al. 2013). National strategy of Vietnam also emphasises the importance of regional planning for SWM (PMSRV 2018). Additionally, planners must also consider carefully the local conditions to have a proper SWM plan (Oteng-Ababio et al. 2017). However, most of the published social studies—Vietnam and other developing countries—have not focused on both regional level and local conditions at the same time. For example, a study—examining the driving factors of WSS of households—only focused on one ward and two communes in Hoi An City, Vietnam (Loan et al. 2017). In Hanoi City of Vietnam, a study used TPB to identify factors affecting WSI in residential areas of Nguyen Du ward (Nhung 2023) while another study applied econometric analysis to depict factors giving an explanation to residential intention towards WSS in four wards including Nguyen Du ward (Phuong et al. 2015). Similarly, two studies in Malaysia examined driving forces of FSI but focused only on Miri City (Ng et al. 2021) and Universiti Putra Malaysia (Ghani et al. 2013).

This study was an effort to fill literature gap and accelerate effectiveness of municipal SWM in Vietnam—a developing country—for some reasons. Firstly, this study extended TPB by adding AB, IP, SF, FA, and trust to original model, and applied it to analyse influencing factors of FSI among households in central Vietnam. Secondly, this study was conducted in three typical types of municipalities in Vietnam, being a foremost effort to collect insights into the driving forces of FSI both at regional and local levels. Moreover, the authors also analyse the heterogeneity of relationships of factors contributing to FSI between three study sites. Finally, geographic information system (GIS) was applied to ensure sampling frame for random sampling. The results of this study will offer scientific evidence for enhancing household WSS and promoting system of SWM, being precursor for paving the way to CE in developing countries.

## Theoretical framework and development of hypotheses

### Theory of planned behaviour

The theory of reasoned action (TRA) was published in the 1970s and is acknowledged as the forebear of TPB (Fishbein and Ajzen 1975). The transformation from the TRA to the TPB featured an expansion of TRA via addition of perceived behavioural control (PBC), thus the belief of people that they can control their actions, as well as an indicator of “available resources and opportunities” (Ajzen and Madden 1986). However, as human behaviour is complex, TPB alone cannot fully explain the forces driving the behaviours of waste generators (Tang et al. 2022; Tonglet et al. 2004). This study used an extended version of the TPB to acquire an understanding of FSI among households in central Vietnam.

### Attitude

Attitude is one of the three independent determinants of intention, and refers to human evaluation and appraisal of a specific behaviour (Ajzen 1991). Recently, attitude has been shown to be positively correlated with FSI (Ghani et al. 2013; Oehman et al. 2022), WSI by household residents (Nhung 2023; Tang et al. 2023), and food-waste reduction intention (Chun T’ing et al. 2021). Positive attitudes would be expected to encourage FSI.

- H1: A positive correlation would be apparent between attitude and the behavioural intentions of households in central Vietnam towards FSS.

### Subjective norm

A subjective norm (SN) is social pressure towards a particular behaviour, being antecedent to human intention towards a behaviour (Ajzen 1991). The SN was shown to be positively associated with FSI (Ghani et al. 2013; Oehman et al. 2022) and WSI (Fan et al. 2019; Nhung 2023; Tang et al. 2023). Moreover, SN also correlated with food-waste reduction intention (Chun T’ing et al. 2021), disposal intention (Dhanabalan et al. 2023), and intention of leaving no waste when hiking (Ng 2023). Thereby, SN was anticipated to positively correlate with FSI.

- H2: A positive relationship would be apparent between SN and the behavioural intentions of households in central Vietnam towards FSS.

### Perceived behavioural control

The PBC is level of difficulty encountered when engaging in a behaviour (Ajzen 1991). The PBC was shown to be positively associated with household FSI (Ghani et al. 2013), household WSI (Oehman et al. 2022; Tang et al. 2023), and food-waste reduction intention (Chun T’ing et al. 2021). Hence, PBC was anticipated to positively correlate with FSI.

- H3: A positive relationship would be apparent between PBC and the behavioural intentions of households in central Vietnam towards FSS

### Awareness of benefit

Awareness of benefits (AB) could be acknowledged that FSS would have a good impact on the environment and human life (Adu-Gyamfi et al. 2023; Phuong et al. 2015). Existing studies proved that awareness of environmentally friendly actions promotes WSI (Adu-Gyamfi et al. 2023; Cudjoe et al. 2020). Therefore, AB was anticipated to positively correlate with FSI.

- H4: A positive relationship would be apparent between AB and the behavioural intentions of households in central Vietnam towards FSS.

### Information publicity

Information publicity (IP) includes public activities scheduled by authorities and information circulated by such authorities to achieve policy objectives via a media campaign (Si et al. 2022). In this study, information refers to data or guidance on FSS. Positive associations between such IP and household WSI have been noted (Li et al. 2023; Tang et al. 2023; Wang et al. 2019). Thus, IP was anticipated to positively correlate with FSI.

- H5: A positive relationship would be apparent between IP and the behavioural intentions of households in central Vietnam towards FSS.

### Situational factor

Situational factors (SF) such as amount of effort required, convenience, storage space, and access to various schemes affect pro-environmental behaviours (Boldero 1995). The lack of knowledge and time was also proved to be significantly related to household WSI (Phuong et al. 2015; Wang et al. 2020). Thus, SF would positively influence FSI.

- H6: A negative relationship would be apparent between SF and the behavioural intentions of households in central Vietnam towards FSS.

### Facility availability

Inadequate facilities pose key challenges to pro-environmental behaviour (Tangwanichagapong et al. 2017). The success of SWM is critically dependent on basic infrastructure such as sorting equipment, quality service, and allied infrastructure (Bernstad 2014; Rispo et al. 2015). Facility availability (FA)—availability of waste bins, separate collection, collection stations, and composter units—was denoted to directly influence FSI (Ng et al. 2021). Therefore, FA positively influences FSI.

- H7: A positive relationship would be apparent between FA and the behavioural intentions of households in central Vietnam towards FSS.

### Trust

Trust is the willingness of a party to act based on a hope that another will act is a way that is meaningful to the trustor (Mayer et al. 1995). Here, trust can be defined as a household willingness to separate food waste based on the expectation that all stakeholders will do so. Trust was a key in terms of household WSI (Phuong et al. 2015).

- H8: A positive relationship would be apparent between trust and the behavioural intentions of households in central Vietnam towards FSS.

### Research model

**Fig. 1:**
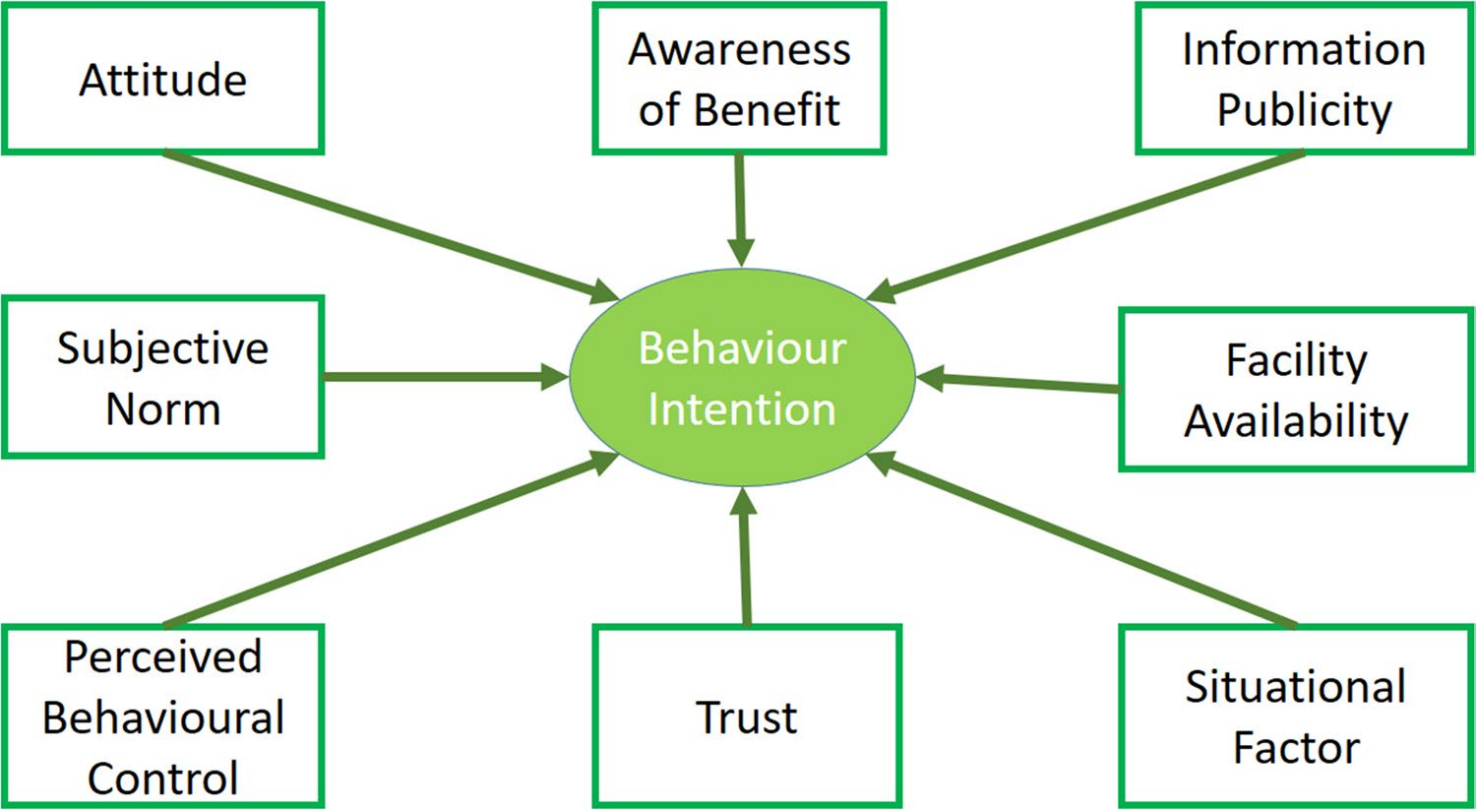
The research model

Using the various hypotheses, the model applied in this study is shown in Fig. 1. There are nine latent variables. The model indicators are listed in Supporting Information (SI) Table A1.

## Methodology

### Study sites

**Fig. 2:**
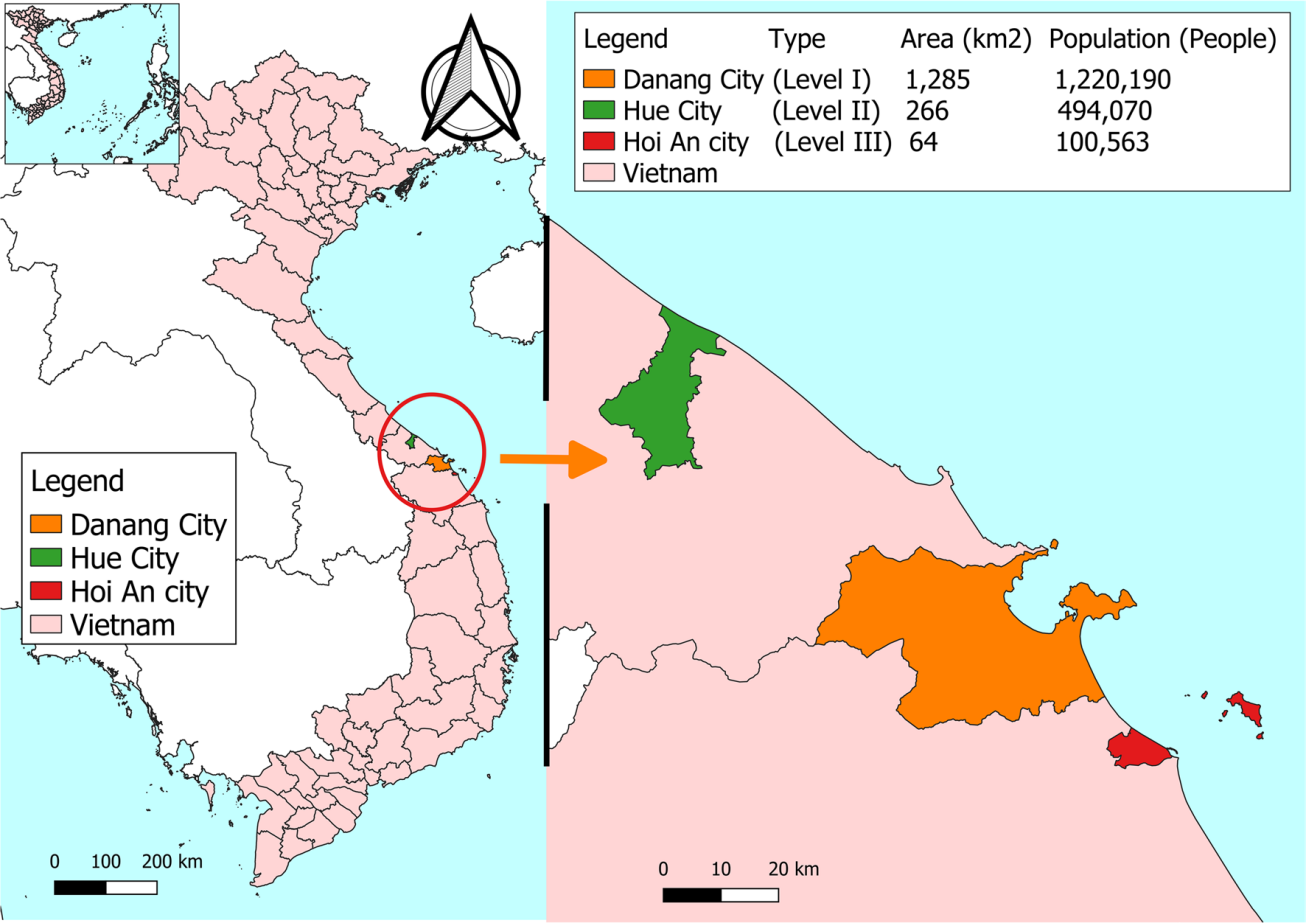
Map of the study sites in central Vietnam

This study was conducted in central Vietnam, where SWM procedures vary significantly. Households of Danang City (level I), Hue City (level II), and Hoi An City (level III) were participants in this study. Populations of Danang, Hue, and Hoi An were approximately 1.2 million, 0.5 million, and 0.1 million people while areas of these cities were 1,285 km^2^, 266 km^2^, and 64 km^2^ (Fig. 2). These three cities represent three distinct types of Vietnamese municipalities (NASC 2016). Thus, the analysis and discussion assume that all Vietnamese cities are of one of these three types.

### Survey design and procedures

#### Questionnaire development

A questionnaire with both closed- and open-ended questions was developed via literature review and consultation with specialists well versed in SWM in central Vietnam. Thus, both quantitative data and qualitative information was collected. Measurement items for constructs—AB (Adu-Gyamfi et al. 2023; Phuong et al. 2015; Zhang et al. 2018), IP (Li et al. 2023; Tang et al. 2023; Wang et al. 2019), SF (Ghani et al. 2013; Wang et al. 2020), FA (Ng et al. 2021), and trust (Loan et al. 2017; Nhung 2023; Phuong et al. 2015)—were adapted and extracted from these mentioned studies. Participants self-evaluated their agreement with statement in items ultilised with seven-point Likert scale, ranging from 1 (strongly disagree) to 7 (strongly agree).

#### Data collection

This survey was conducted by a team from the University of Technology and Education, The University of Danang. The survey was conducted in two main stages: stage 1 (the pre-survey) from February to March 2023 and stage 2 from July to August of 2023. In stage 1, a presurvey was conducted among 20 students and their households to detach ambiguous meanings or incorrect expressions within local context. Thus, minor modifications, were proceeded basing on the feedback of the presurvey. In stage 2, data collection was conducted aimed at fulfilling requirements from sample size for data analysis. Participants—households in three cities (Danang, Hue, and Hoi An)—were randomly sampled to avoid bias or discrimination. This study applied GIS to solve problem of unavailability of sampling frame for probability sampling. After acquiring GIS data on locations of households, participants (households) were randomly chosen within GIS environment. Members of survey team visited chosen household and invited representative people of household to answer questionnaire. We focused only on family members (being representative for household) older than 18 years who were responsible for waste management.

Finally, a total of 997 questionnaires were collected, of which 976 were valid and 21 not. Invalidity included linear/diagonal responses to all questions, age under 18 years, and an uncooperative attitude. The sample sizes were 409, 260, and 307 in Danang, Hue, and Hoi An, respectively. The sample sizes for each municipal type met the minimum sample sizes of the guidelines from Hair et al. (2017) and Cohen (1992).

### Data analysis

In general, there are four main stages of data analysis. Firstly, common method bias (CMB) was checked. Secondly, the outer model (the measurement model) was assessed based on outer loadings, Cronbach’s alpha, composite reliability, average variance extracted (AVE), and heterotrait-monotrait ratio (HTMT). Moreover, bootstrapping was also applied to check HTMT value in this stage. Thirdly, the inner model (the structural model) was tested including multicollinearity check and bootstrapping analysis. The procedures for assessment of the outer and inner models were those of Hair et al. (2017, 2019). Hypothesis testing of the path coefficients of the models for all three cities featured bootstrapping using 5000 subsamples. Finally, multigroup analysis (MGA) was utilised to depict the differences in relationships of factors contributing to FSI between three levels of cities in central Vietnam. Before MGA, the measurement invariance of the composite models (MICOM) test is standard; this checks for measurement invariance (Henseler et al. 2016). Moreover, the *p*-value of multigroup analysis was modified using the Šidák and Bonferroni adjustment (Cheah et al. 2023).

Descriptive analysis employed Microsoft Excel software. SmatPLS 4 was used to check for common method bias, and to examine and analyse the outer model, inner model, and the MGA (Ringle et al. 2022).

## Result

### Descriptive analysis

**Fig. 3:**
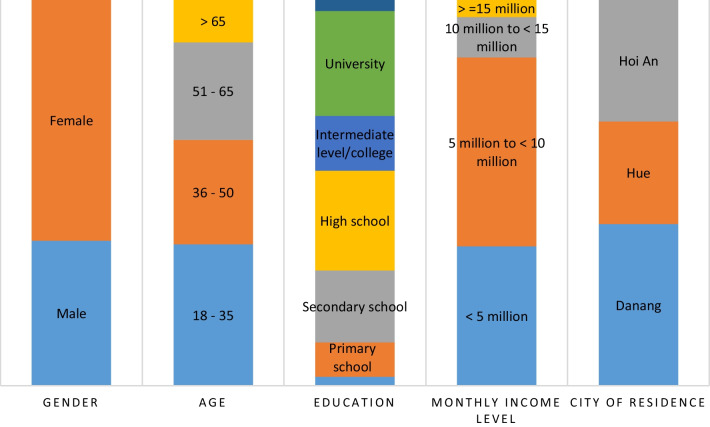
Demographic information of respondents

The total sample number was 976, 37.6% males and 62.4% females. The explanation for the high percentage of female participants compared with male participants would be females were mainly in charge of food-waste disposal in central Vietnam. Most respondents were aged 18–35 years (36.7%) followed by 36–50 years (27%), 51–65 years (25.3%), and > 65 years (11%) (Fig. 3). The age structure of sample was corresponding to age structure of Vietnam, with the highest proportion of people aged 18–35 years (GSOV 2021). The proportion of people being older than 65 years old is lowest at 11%, being similar to proportion of old people of Vietnam (MLWS 2023).

In terms of educational level, those with university and high school education constituted 27.2% and 25.8% of all respondents, respectively, followed by those with secondary school (18.6%), intermediate level/college (14.2%), and primary school (8.9%) education. Noticeably, illiterate and postgraduate respondents accounted for 2.4% and 2.9% of all respondents, respectively (Fig. 3). The proportion of people with intermediate level/college and above was high, at 44.3% compared with mean value of whole country (19.3%) (GSOV 2022). This study was conducted in three cities; thus, the education level would be expected to be higher, especially in comparison with rural areas (An and Huong 2017; GSOV 2021).

Most respondents (49%) had monthly incomes between 5 million VND (205 USD) and 10 million VND (410 USD). Notably, 36.2% of respondents had monthly incomes of less than 5 million VND (205 USD). Most of respondents’ income (85%) fluctuated around the average income (about 5 million VND) per capita of Vietnam (GSOV 2022). Respondents with monthly incomes between 10 million VND (410 USD) and 15 million VND (615 USD) accounted for 10.5% of the total. People with high monthly incomes (more than 15 million VND [615 USD]) accounted for only 4.4% (Fig. 3). The proportion of people had income, being far to national average income level, was low at about 15%. These phenomena denoted the proper sampling for analysis regarding income level.

### Common method bias (CBM)

The VIF values of all constructs were lower than 3.3 (Table 1), indicating the absence of CBM (Kock 2015; Kock and Lynn 2012).

**Table 1:** Results of the common bias method test

	Central Vietnam	Level I	Level II	Level III
Attitude—> random variable	1.341	1.649	1.922	1.664
Awareness of benefit—> random variable	1.041	1.764	2.206	1.470
Behaviour intention—> random variable	1.028	1.826	1.586	1.080
Information publicity—> random variable	1.100	1.682	1.110	1.795
Facility availability—> random variable	1.029	1.208	1.146	1.090
Perceived behaviour control—> random variable	1.055	1.041	1.367	1.757
Situational factor—> random variable	1.068	1.082	1.068	1.137
Subjective norm—> random variable	1.136	1.532	1.654	1.548
Trust—> random variable	1.278	1.231	1.268	1.645

#### Outer model evaluation

Model assessment followed the guidelines of Hair et al. (2019) and Hair et al. (2017). The results of outer model evaluation are shown in Supporting Information (SI) Tables B1–B14. All indicators of outer model evaluation including the outer loading, Cronbach alpha, composite reliability, average variance extracted (AVE), and the Heterotrait-Monotrait ratio of correlation (HTMT) satisfied the conditions required for further data analysis.

### Evaluation of the structural model (inner model)

The VIF values of all latent variables, thus central Vietnam, and the level I, level II, and level III cities, were lower than the threshold of 5 (SI Table C1); multicollinearity was thus absent (Hair et al. 2019).

**Fig. 4:**
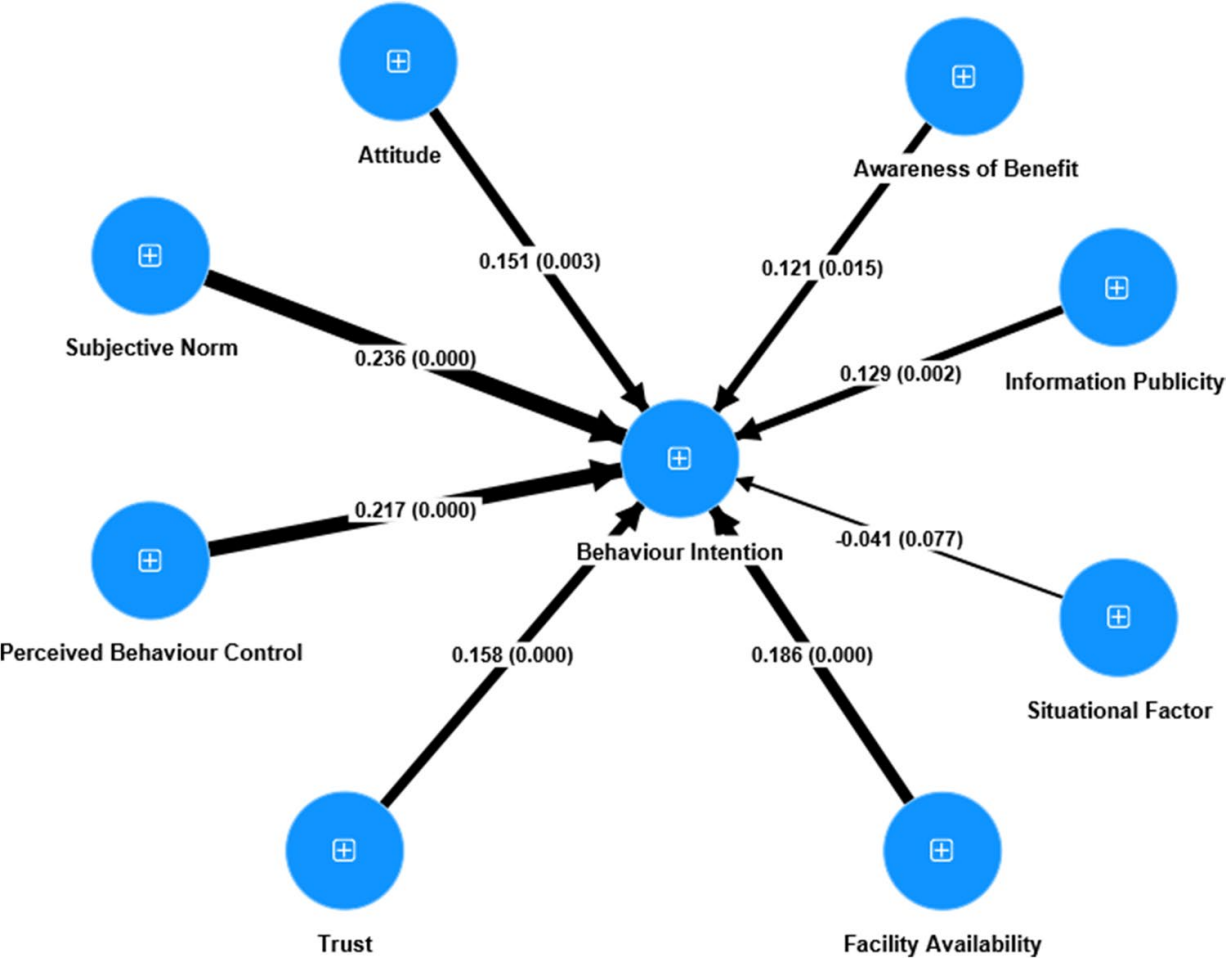
PLS-SEM structural model for central Vietnam

In central Vietnam, the results indicated that all latent variables had significant positive impacts on FSI (*p*-values < 0.05), with the exception of SF (Fig. 4; SI Table C2). Specifically, SN exhibited the highest *β* value (0.236), followed by PBC (0.217), and FA (0.186). The *β* values for attitude, AB, IP, and trust varied between 0.121 and 0.158, and were thus much lower than those for SN and PBC (Fig. 4; SI Table C2). The *p*-values for SF were 0.077, thus larger than 0.05. This means that SF were not significantly related to FSI (Fig. 4; SI Table C2).

### Multigroup analysis

The permutation *p*-values of all latent variables among the three cities were all greater than or equal to the 1% quantile (Tables 2, 3, and 4). Thus, compositional invariance was in play (Henseler et al. 2016). Thus, the MGA conditions were fulfilled and a comparison of path coefficients among the cities (groups) was appropriate (Cheah et al. 2020).

**Table 2:** MICOM step 2: composition invariance (level I vs. level III cities)

Level I vs. level III					
	Original correlation	Correlation permutation mean	1.00%	Permutation *p* value	Compositional Invariance?
Attitude	1.000	1.000	0.998	0.375	Yes
Awareness of benefit	0.992	0.997	0.982	0.081	Yes
Behaviour intention	0.999	1.000	0.998	0.102	Yes
Facility availability	0.999	0.999	0.995	0.519	Yes
Information publicity	1.000	0.891	0.038	0.984	Yes
Perceived behaviour control	0.997	0.999	0.995	0.106	Yes
Situational factor	0.880	0.954	0.033	0.071	Yes
Subjective norm	0.998	0.994	0.949	0.640	Yes
Trust	0.998	0.998	0.994	0.344	Yes

**Table 3:** MICOM step 2: composition invariance (level I vs. level II cities)

Level I vs. level II					
	Original correlation	Correlation permutation mean	1.00%	Permutation *p* value	Compositional invariance?
Attitude	1.000	1.000	0.998	0.198	Yes
Awareness of benefit	1.000	0.999	0.99	0.572	Yes
Behaviour intention	1.000	0.999	0.997	0.694	Yes
Facility availability	0.998	0.999	0.994	0.188	Yes
Information publicity	1.000	0.997	0.983	0.848	Yes
Perceived behaviour control	1.000	0.999	0.994	0.716	Yes
Situational factor	1.000	0.998	0.987	0.702	Yes
Subjective norm	0.999	0.999	0.996	0.469	Yes
Trust	0.998	0.999	0.994	0.227	Yes

**Table 4:** MICOM step 2: composition invariance (level II vs. level III cities)

Level II vs. level III					
	Original correlation	Correlation permutation mean	1.00%	Permutation *p* value	Compositional Invariance?
Attitude	0.999	0.999	0.993	0.316	Yes
Awareness of benefit	0.989	0.997	0.98	0.056	Yes
Behaviour intention	0.998	0.999	0.995	0.2	Yes
Facility availability	0.998	0.997	0.987	0.528	Yes
Information publicity	1	0.885	-0.211	0.991	Yes
Perceived behaviour control	0.998	0.999	0.994	0.158	Yes
Situational factor	0.887	0.936	-0.163	0.109	Yes
Subjective norm	0.999	0.993	0.943	0.731	Yes
Trust	0.996	0.996	0.985	0.372	Yes

The original mean and variance differences did not completely fall within the 1% and 99% boundaries (SI Tables D1-D3). Thus, full measurement invariance could not be established; partial measurement invariance was identified (Henseler et al. 2016). As a result, the data should be analysed by group (the city levels) (Henseler et al. 2016).

The MGA results when level I and level III cities were compared indicated significant differences in terms of the relationship between attitude and FSI (difference = 0.304; *p*-value = 0.004) and between SF and FSI (difference = – 0.278; *p*-value = 0.016) (Table 5). A significant difference between level I and level II cities was found in terms of the linkage between PBC and FSI (difference = 0.246, *p*-value = 0.011) (Table 5). The MGA analyses thus also indicated a significant difference (difference = – 0.346, *p*-value = 0.001) in terms of the direct effect of PBC on FSI between level II cities and level III cities (Table 5).

**Table 5:** The results of multigroup analysis by the levels of cities in central Vietnam

	Level I–level III			Level I–level II			Level III–level II		
	Difference	*P* value	Conclusion	Difference	*P* value	Conclusion	Difference	*P* value	Conclusion
Attitude—> behaviour intention	0.304	0.004	Supported	0.096	0.39	Not	0.208	0.069	Not
Awareness of benefit—> behaviour intention	− 0.092	0.375	Not	− 0.021	0.864	Not	− 0.071	0.611	Not
Facility availability—> behaviour intention	− 0.122	0.201	Not	− 0.088	0.327	Not	− 0.034	0.75	Not
Information publicity—> behaviour intention	0.19	0.074	Not	0.134	0.123	Not	0.057	0.615	Not
Perceived behaviour control—> behaviour intention	− 0.101	0.276	Not	0.246	0.011	Supported	− 0.346	0.001	Supported
Situational factor—> behaviour intention	− 0.278	0.016	Supported	0.014	0.819	Not	− 0.292	0.018	Not
Subjective norm—> behaviour intention	− 0.022	0.752	Not	− 0.109	0.2	Not	0.086	0.356	Not
Trust—> behaviour intention	0.186	0.04	Not	0.067	0.409	Not	0.119	0.184	Not

## Discussion

### The results for central Vietnam

#### Attitude

In central Vietnam, attitude statistically contributed to FSI (Fig. 4). Similarly, attitude positively affected FSI among staff of a university in Malaysia (Ghani et al. 2013) and American households (Oehman et al. 2022), and food-waste reduction intention among Malaysians (Chun T'ing et al. 2021), and WSI among residents of Ghana (Tang et al. 2023) and Hanoi City, Vietnam (Nhung 2023). This indicates that attitudes towards FSI play an important role in combating waste-related problems, especially by enhancing FSI in central Vietnam. Despite the importance of attitude to enhancing FSI, the improvement of pro-environmental attitude in developing countries would be a challenge due to low level of education (Kremer et al. 2013) and living standards (Egger et al. 2021). Thus, the enhancement of FSI through shift in people’s attitude should be considered in a comprehensive manner with education and economic development.

#### Subjective norm

The SN significantly affected FSI in central Vietnam (Fig. 4). This result aligns with those of many studies indicating positive effects of SN on behavioural intentions in terms of FSI (Ghani et al. 2013; Oehman et al. 2022), WSI (Fan et al. 2019; Nhung 2023; Tang et al. 2023), disposal intention (Dhanabalan et al. 2023), and intentions to leave no trace when hiking (Ng 2023). These results indicate that the positive effects of family, friends, or colleagues on FSI should be considered when developing policies and strategies for food-waste management (FWM). Humans are social animals (Aronson 1972; Tomasello 2014); thus, it is inevitable that people’s psychology, behaviours, and thought are strongly affected by social environment (Cohen et al. 2021). Thus, foundation and development of an environmentally friendly community would also be proper approach for enhancing FSI. In central Vietnam, these communities operating by government-related women’s union and youth union have presented effectiveness in recyclable waste separation and other pro-environmental activities (UNESCAP 2021).

#### Perceived behaviour control

The results denoted the importance of such PBC in terms of FSI in central Vietnam (Fig. 4). The importance of PBC towards FSI and WSI was also identified in several studies in America (Oehman et al. 2022), Ghana (Tang et al. 2023), and Malaysia (Ghani et al. 2013). Greater PBC increases FSI, followed by rises in the efficiency and effectiveness of FSS. It should be noted that the perception of humans is strongly dependent on context of environment (Chinazzo et al. 2019; Johnson 2021; Rita 1994; Wu et al. 2020, 2022). The human perception would also be a result of a long history of interaction with external environment as well as future goals (Johnson 2021). Thus, the remediation of FSI through PBC would be conducted with long-term-strategy thinking in a comprehensive manner to cover past experience, present context, and expected goals through education, propaganda, and technical solution. For example, in Japan, the success of solid waste separation program (SWSP) is result of long-term, continuous, and comprehensive strategies and activities (CECJ 2010). Importance of long-term and comprehensive thinking of planners and managers is emphasised.

#### Awareness of benefit

The AB had significant impact on FSI in central Vietnam (Fig. 4). This result is in line with the findings of studies denoting AB exhibited significant relationship with WSI (Adu-Gyamfi et al. 2023; Cudjoe et al. 2020). This indicates that citizens consider environmental benefits such as the reduction of waste to landfills and the educational role for the next generations afforded by FSS as significant drivers of FSI. Thus, the promulgation of environmental and educational benefit of FSS should be promoted with expectation of positive impact on FSI. However, in developing countries, the effectiveness of promulgation normally not meet requirements of planners or managers due to lack of resource for continuous implementation, gap between content of promulgation, and realistic results, as well as unsystematic implementation of promulgation and other social-technical issues (Linda et al. 2019). The systematic, continuous, and long-term implementation of SWM measures has remained a challenge for not only FSS but also other pro-environmental activities in developing countries.

#### Information publicity

The IP was positively correlated with FSI in central Vietnam (Fig. 4). This result aligns with that of a study reporting that IP positively affected residents’ WSI in China (Wang et al. 2019), Pakistan (Li et al. 2023), and Ghana (Tang et al. 2023). In central Vietnam, it is important that the majority of people can readily access information on FSS, in turn enhancing FWM. Recently, given the boom in digital transformation, information can be delivered very effectively and with minimal effort via a variety of social networks. The IP should be more widely dispersed in central Vietnam, especially in the context of Industry 4.0. Despite effortless and rapid spreading of information on environmental campaign, people tend to focus on trending topics rather than information on the environment (Yang and Peng 2020). Thus, ultilisation of social networks for spreading information on pro-environmental activities—FSS—would be considered in competition with trending topics. Normally, human and economic resources spent on pro-environmental activities are limited compared with others such as e-commerce. Thus, the ultilisation of social network and media should be considered carefully to have the highest effectiveness.

#### Situational factor

The effects of SF on FSI were not significant in central Vietnam (Fig. 4). This result is similar to that of studies in Malaysia (Ghani et al. 2013), which reported an insignificant relationship between SF and recycling intentions. Thus, time and knowledge were not important in terms of FSS in central Vietnam. Most respondents thought that the additional time required to FSS was acceptable. The skill and knowledge required for FSS were thought to be low. If the inner motivation is strong enough, people would themselves have pro-environmental intention despite extrinsic difficulties, namely lack of knowledge or time (Schwartz et al. 2015). Challenge is not the external environment but the enhancement of inner motivation of human beings for FSI and FSS.

#### Facility availability

In central Vietnam, FA correlated with FSI (Fig. 4), indicating a meaningful statistical relationship. This finding is consistent with a study in Malaysia, which found that FA influenced the FSI of those who created food waste (Ng et al. 2021). Thus, the building of facilities for FSS is extremely important; this is key in terms of the behavioural intentions of waste generators. Moreover, NIMBY (not in my back yard) is still a popular thinking among people so that the availability of facilities for FSS plays a preeminent role in enhancing pro-environmental intention and behaviour (Holm et al. 2021; Simsek et al. 2014). Methods of food-waste collection (bins, transfer stations, or types of service providing) and foundation of facilities for FSS should be carefully planned before introducing FSS plans at regional and local levels. In areas of high urbanisation and high density of economic activities, the foundation of SWM facilities will be limited due to priority for urban aesthetics and economic-driven activities while, in rural areas, the foundation of facilities would be easier due to the availability of unused spaces. Moreover, in developing and poorly developed countries, initial investment in food-waste treatment facilities is challenging; SWM budgets are low. The international support of non-governmental organisations (NGOs) plays key roles in such countries.

#### Trust

Trust was significantly and positively associated with FSI in central Vietnam (Fig. 4). This result is similar to that of a study in Hanoi, Vietnam, showing that trust significantly influenced the WSI of households (Phuong et al. 2015). It is understandable that trust would enhance the intentions of citizens to separate food waste at source. The importance of trust was also emphasised in a study on SWM in central Vietnam (Song Toan et al. 2019). If the food-waste handling system is not trusted by waste generators, the effectiveness and efficiency of a FSS programme will be negatively affected. Despite positive relationship between trust and FWI, trust of people for SWSP would be low due to negative results from SWSP of developing countries (Cuong et al. 2021). Thus, enhancement of trust for SWSP would be a big challenge for developing countries. However, publicity and transparency are argued to be strongest drivers to improve citizens’ trust (Edwards 2020; Sofyani et al. 2022). Thus, publicity and transparency from SWSPs, collection, and transportation, as well as final treatment will plays a pivotal in enhancing pro-environmental program—FSS.

### MGA

The effect of PBC on FSI was lower in the level II city than in the level I and level III cities (Table 5). The human personality would be the reason for these differences (Lewin and Sager 2013; Liu et al. 2016). Hue (level II) is the former capital of Vietnam where traditional thinking remains in play. Independent decisions are not common, or sometimes, constrained (PCTTHP 2020). On the other hand, Danang is a “young city” with vibrant economic development and Hoi An is a tourism destination with millions of annual international arrivals. People of Danang (level I) and Hoi An (level III) are also considered friendly and open minded (Hien Nguyen 2018). Thus, the decision-making would be more independent compared with Hue City (level II). As a result, the differences in human personality would be the reason for differences in the effect of PBC on FSI in Hue City (level II) compared with other cities (level I and level III).

The difference in the impact of attitude to FSI between level I and level III cities was significant (Table 5). This may indicate that citizen attitudes of concern towards the environment (FSI) were significantly higher in the level I city than in the level III city. Level I cities are the largest municipalities of Vietnam, and play key roles in the economic development of central Vietnam. It is thus understandable that citizens’ attitudes of concern in level I cities would be higher than those of citizens in level III cities (Satterthwaite 1997). Thus, FWM policies must consider these attitudinal differences. For example, in level III cities, policy should focus on the development of positive attitudes towards FWM; promotion of established management policies would be more appropriate in level I cities.

Through the results of MGA, it can be concluded that despite the significant influence of factors (PBC and attitude) on FSI at the regional level (central Vietnam), the effects of these factors on FSI are different among municipal levels. Thus, it is a need to consider both regional views and local conditions to conduct SWM planning to enhance FSS, being a precondition to promote CE (Ilić and Nikolić 2016).

### Implication

This study applied an extended version of TPB to detach factors influencing food waste separation in central Vietnam. Added variables such as AB, IP, FA, and trust asserted positive relationships to FSI. Thus, this paper offered a comprehension of varied factors contributing to FSI compared with studies that applied original TPB.

This study also applied GIS to solve problem of unavailability of sampling frame, especially in developing countries, for random sampling (Chun T’ing et al. 2021; Kiregyera 1982; Reichel and Morales 2017). As a result, this study took advantage of previous SWM studies which apply non-probability sampling such as convenient sampling (Pham et al. 2023), purposive sampling (Li et al. 2023), quota sampling (Chun T’ing et al. 2021) whose results would have limited representativeness—only cover specific study areas. Moreover, this study could also have some advantages in comparison to other studies that applied probability sampling but through online platform (Adu-Gyamfi et al. 2023; Oehman et al. 2022; Tang et al. 2023; Wang et al. 2019). In developing countries, the access and normal use of social networks would be limited to a proportion of citizens; thus, representativeness for the whole population should be questioned (Van Selm and Jankowski 2006). Moreover, the seriousness in response to online questionnaires should be questioned, leading to challenges in quality control of responses. Thanks to GIS support for sampling, this study would have representativeness being base for regional and local planning of SWM not only in Vietnam but also in other developing countries.

Notably, the application of results from this study to enhance FSI and FSS should be carefully considered due to limitation of resources for pro-environmental activities in developing countries. The detailed discussion of problems and challenges has been proposed to specific factors influencing FSI. Thus, the results of this study were expected to serve as a comprehensive scientific base for planners and SWM officers in designing the food waste management strategy—heading towards CE—in developing countries which have tantamount features to Vietnam.

## Conclusion

This study clarified underlying factors affecting FSI by applying extended TPB in central Vietnam. These include attitudes, SN, PBC, AB, IP, FA, and trust. In addition, heterogeneity in relationships of factors contributing to FSI between levels of cities in central Vietnam was revealed via MGA. This proved that despite general features of factors affecting FSI in central Vietnam, the effect of these factors on FSI in varied municipal levels would be different. This study also contributed as an effort to solve problem of unavailability of sampling frame for random sampling by applied GIS. However, this solution needs a proper amount data for the whole study sites and GIS skill. Thus, future social studies could consider the multidisciplinary cooperation to solve not only problems of sampling frame but also other related problems in social research.

This study was cross-sectional in nature. However, the world is changing rapidly; future studies should consider such changes by integrating longitudinal data. Also, future studies should seek to have similarities in the group sample sizes to enhance MGA results. Moreover, due to the complexity of mechanism of human behaviour, the measure of actual behaviour and integration of a variety of social theories would be promoted in future social studies on food waste management.

The results of this study will facilitate FSS, supporting establishment of a CE and being a reference for policymakers, planners, NGOs, and other related stakeholders who promote a CE in Vietnam and other developing countries. Moreover, results imply appropriate regional policies and strategies that consider local characteristics, ensuring effective and efficient FWM not only in Vietnam but also other developing countries.

### Supplementary Information

Below is the link to the electronic supplementary material.

Supplementary file1 (DOCX 51.8 KB)

## Data Availability

The detailed results of analysis are included in Supporting Information of this paper. The other data will be available from the authors upon reasonable request and with the permission of related parties.
