# Supplementary material for: Understanding driving forces of food waste separation intention to enhance regional and local solid waste planning: application of PLS-SEM and multi-group analysis
Source: Environ Sci Pollut Res Int. 2024 Aug 5;31(38):50654–69. doi: 10.1007/s11356-024-34485-2 (PMC11364603; doi:10.1007/s11356-024-34485-2)
Supplement: Supplementary file 1 — Supplementary file1 (DOCX 51.8 KB) [file 11356_2024_34485_MOESM1_ESM.docx]

# Supporting Information

# A. Indicator of model

Table A1. Indicator of model

| **Variable** | **Items** | **Description of Items** |
| --- | --- | --- |
| Dependent variable: Behavioral Intention (BI) | BI1 | 1. I intend to separate my food waste at home on a regular basis if there are food waste collection measures |
|  | BI2 | 2. I plan to participate in food waste separation at home if I am satisfied with the food waste collection measures by the local authorities |
|  | BI3 | 3. I will try my best to separate my food waste at home if am convinced with the benefits of food waste separation at home |
|  | BI4 | 4. I will make an effort to separate my food waste at home if the local authority enforces public participation in food waste separation at homes |
|  | BI5 | 5. How much of your HFW do you plan to separate in the next 12 months? (none, all) |
|  | BI6 | 6. I am determined to separate HFW in the next 12 months (does not describe me, completely describes me) |
| Independent variable: Attitude (AT) | AT1 | 1. I think food waste separation should be further promoted in Vietnam |
|  | AT2 | 2. I think separating my food waste at home is needed |
|  | AT3 | 3. I think food waste separation is good for environment |
| Independent variable: Awareness of Benefits (AB) | AB1 | 1. I believe that waste separation helps reduce disposal of waste in landfills |
|  | AB2 | 2. I think that my waste separation behavior will have an important educational effect on my children |
| Independent variable: Information Publicity (IP) | LP1 | 1. I think the information publicized through social network about waste separation is important |
|  | LP2 | 2. I think the information publicized through television about waste separation is important |
|  | LP3 | 3. I think the information publicized through community meeting about waste separation is important |
| Independent variable: Situational Factor (SF) | SF1 | 1. I think not having enough time to properly separate my food waste would be difficult for me to separate my food waste at home |
|  | SF2 | 2. I think not having proper knowledge on how to separate my food waste would make it difficult for me to separate my food waste at home |
| Independent variable: Facility Availability (FA) | FA1 | 1. I think separating collection of food waste will encourage my participation |
|  | FA2 | 2. I think provision of food waste bins will encourage my participation |
|  | FA3 | 3. I think food waste collection centers being set up in neighborhood will encourage my participation |
|  | FA4 | 4. I think provision of a composter unit to compost our own food waste will encourage my participation |
| Independent variable: Trust (TRU) | TRU1 | 1. I trust local authority has ability to treat sorted waste |
|  | TRU2 | 2. I trust local authority strictly implement punishment methods for non-participants |
|  | TRU3 | 3. I trust that the government, with effective legal instruments, has been making efforts in waste management |
|  | TRU4 | 4. I trust that other members in my community will comply with the regulation of waste separation at source |
| Independent variable: Perceived Behavioural Control (PBC) | PBC1 | 1. I think decision to separate my food waste is completely up to me |
|  | PBC2 | 2. I think separating food waste at home would be an easy task |
|  | PBC3 | 3. I think I have complete control in deciding whether or not to separate my food waste at home |
|  | PBC4 | 4. I have the feeling that I can do something about the food that I waste |
|  | PBC5 | 5. I think I have ability to separate food waste |
| Independent variable: Subjective Norm (SN) | SN1 | 1. My family would think I should separate my food waste at home |
|  | SN2 | 2. My neighbors would think I should participate in food waste separation at home |
|  | SN3 | 3. The community in the area I live think I should separate my food waste at home |

# B. Outer model analysis

Table B1. Outer loading of indicator

|  | Central Vietnam | Danang (Level I) | Hue (Level II) | Hoi An (Level III) |  | Central Vietnam | Danang (Level I) | Hue (Level II) | Hoi An (Level III) | |
| --- | --- | --- | --- | --- | --- | --- | --- | --- | --- | --- |
| AB1 <- Awareness of Benefit | 0.894 | 0.879 | 0.895 | 0.923 | **LP2 <- Information publicity** | 0.952 | 0.898 | 0.886 | 0.863 |  |
| AB2 <- Awareness of Benefit | 0.858 | 0.866 | 0.908 | 0.773 | **LP3 <- Information publicity** | 0.946 | 0.868 | 0.854 | 0.871 |  |
| AT1 <- Attitude | 0.907 | 0.939 | 0.93 | 0.806 | **PBC1 <- Perceived Behaviour Control** | 0.801 | 0.76 | 0.859 | 0.805 |  |
| AT2 <- Attitude | 0.928 | 0.933 | 0.949 | 0.883 | **PBC2 <- Perceived Behaviour Control** | 0.785 | 0.76 | 0.84 | 0.745 |  |
| AT3 <- Attitude | 0.873 | 0.91 | 0.821 | 0.88 | **PBC3 <- Perceived Behaviour Control** | 0.809 | 0.769 | 0.838 | 0.818 |  |
| BI1 <- Behaviour Intention | 0.794 | 0.825 | 0.751 | 0.769 | **PBC4 <- Perceived Behaviour Control** | 0.885 | 0.878 | 0.934 | 0.83 |  |
| BI2 <- Behaviour Intention | 0.82 | 0.823 | 0.777 | 0.838 | **PBC5 <- Perceived Behaviour Control** | 0.844 | 0.852 | 0.905 | 0.723 |  |
| BI3 <- Behaviour Intention | 0.782 | 0.722 | 0.746 | 0.874 | **SF1 <- Situational Factor** | 0.894 | 0.918 | 0.912 | 0.996 |  |
| BI4 <- Behaviour Intention | 0.78 | 0.741 | 0.735 | 0.871 | **SF2 <- Situational Factor** | 0.938 | 0.916 | 0.889 | 0.638 |  |
| BI5 <- Behaviour Intention | 0.792 | 0.788 | 0.812 | 0.789 | **SN1 <- Subjective Norm** | 0.938 | 0.861 | 0.881 | 0.878 |  |
| BI6 <- Behaviour Intention | 0.805 | 0.809 | 0.784 | 0.816 | **SN2 <- Subjective Norm** | 0.954 | 0.919 | 0.916 | 0.826 |  |
| FA1 <- Facility Availability | 0.851 | 0.873 | 0.81 | 0.865 | **SN3 <- Subjective Norm** | 0.934 | 0.835 | 0.854 | 0.897 |  |
| FA2 <- Facility Availability | 0.751 | 0.778 | 0.731 | 0.706 | **TRU1 <- Trust** | 0.792 | 0.827 | 0.777 | 0.744 |  |
| FA3 <- Facility Availability | 0.776 | 0.82 | 0.772 | 0.701 | **TRU2 <- Trust** | 0.766 | 0.763 | 0.784 | 0.74 |  |
| FA4 <- Facility Availability | 0.862 | 0.895 | 0.799 | 0.849 | **TRU3 <- Trust** | 0.814 | 0.797 | 0.818 | 0.841 |  |
| LP1 <- Information publicity | 0.904 | 0.82 | 0.737 | 0.815 | **TRU4 <- Trust** | 0.806 | 0.814 | 0.809 | 0.797 |  |

Table B2. Construct reliability and validity check (Central Vietnam)

| **Central Vietnam** | **Cronbach's alpha** | **Composite reliability (rho_c)** | **Average variance extracted (AVE)** |
| --- | --- | --- | --- |
| **Attitude** | 0.886 | 0.93 | 0.815 |
| **Awareness of Benefit** | 0.698 | 0.868 | 0.767 |
| **Behaviour Intention** | 0.884 | 0.912 | 0.633 |
| **Information publicity** | 0.931 | 0.954 | 0.873 |
| **Facility Availability** | 0.829 | 0.885 | 0.658 |
| **Perceived Behaviour Control** | 0.883 | 0.914 | 0.682 |
| **Situational Factor** | 0.811 | 0.913 | 0.839 |
| **Subjective Norm** | 0.937 | 0.96 | 0.888 |
| **Trust** | 0.805 | 0.873 | 0.631 |

Table B3. Construct reliability and validity check (Danang city)

| **Danang (Leve I)** | **Cronbach's alpha** | **Composite reliability (rho_c)** | **Average variance extracted (AVE)** |
| --- | --- | --- | --- |
| **Attitude** | 0.918 | 0.948 | 0.860 |
| **Awareness of Benefit** | 0.686 | 0.864 | 0.761 |
| **Behaviour Intention** | 0.875 | 0.906 | 0.617 |
| **Information publicity** | 0.836 | 0.897 | 0.744 |
| **Facility Availability** | 0.865 | 0.907 | 0.710 |
| **Perceived Behaviour Control** | 0.864 | 0.902 | 0.649 |
| **Situational Factor** | 0.811 | 0.914 | 0.841 |
| **Subjective Norm** | 0.844 | 0.905 | 0.761 |
| **Trust** | 0.813 | 0.877 | 0.641 |

Table B4. Construct reliability and validity check (Hue city)

| **Hue (Leve II)** | **Cronbach's alpha** | **Composite reliability (rho_c)** | **Average variance extracted (AVE)** |
| --- | --- | --- | --- |
| **Attitude** | 0.884 | 0.929 | 0.813 |
| **Awareness of Benefit** | 0.77 | 0.897 | 0.813 |
| **Behaviour Intention** | 0.862 | 0.896 | 0.590 |
| **Information publicity** | 0.776 | 0.867 | 0.686 |
| **Facility Availability** | 0.789 | 0.860 | 0.606 |
| **Perceived Behaviour Control** | 0.924 | 0.943 | 0.768 |
| **Situational Factor** | 0.769 | 0.896 | 0.812 |
| **Subjective Norm** | 0.862 | 0.915 | 0.781 |
| **Trust** | 0.811 | 0.875 | 0.636 |

Table B5. Construct reliability and validity check (Hoi An city)

| **Hoi An (Level III)** | **Cronbach's alpha** | **Composite reliability (rho_c)** | **Average variance extracted (AVE)** |
| --- | --- | --- | --- |
| **Attitude** | 0.818 | 0.892 | 0.734 |
| **Awareness of Benefit** | 0.638 | 0.839 | 0.724 |
| **Behaviour Intention** | 0.907 | 0.928 | 0.684 |
| **Information publicity** | 0.811 | 0.886 | 0.723 |
| **Facility Availability** | 0.795 | 0.863 | 0.615 |
| **Perceived Behaviour Control** | 0.844 | 0.889 | 0.617 |
| **Situational Factor** | 0.828 | 0.816 | 0.699 |
| **Subjective Norm** | 0.840 | 0.901 | 0.753 |
| **Trust** | 0.787 | 0.862 | 0.611 |

Table B6. Discriminant validity check: Heterotrait-monotrait ratio of correlation (HTMT)

|  | **Central Vietnam** | **Danang (Level I)** | **Hue (Leve II)** | **Hoi An (Level III)** |
| --- | --- | --- | --- | --- |
| **Awareness of Benefit <-> Attitude** | 0.820 | 0.854 | 0.856 | 0.672 |
| **Behaviour Intention <-> Attitude** | 0.593 | 0.753 | 0.626 | 0.406 |
| **Behaviour Intention <-> Awareness of Benefit** | 0.633 | 0.785 | 0.637 | 0.479 |
| **Facility Availability <-> Attitude** | 0.530 | 0.537 | 0.557 | 0.625 |
| **Facility Availability <-> Awareness of Benefit** | 0.572 | 0.656 | 0.528 | 0.542 |
| **Facility Availability <-> Behaviour Intention** | 0.556 | 0.602 | 0.562 | 0.496 |
| **Information publicity <-> Attitude** | 0.103 | 0.518 | 0.455 | 0.606 |
| **Information publicity <-> Awareness of Benefit** | 0.109 | 0.544 | 0.504 | 0.62 |
| **Information publicity <-> Behaviour Intention** | 0.137 | 0.471 | 0.448 | 0.383 |
| **Information publicity <-> Facility Availability** | 0.166 | 0.681 | 0.621 | 0.815 |
| **Perceived Behaviour Control <-> Attitude** | 0.59 | 0.588 | 0.502 | 0.728 |
| **Perceived Behaviour Control <-> Awareness of Benefit** | 0.643 | 0.773 | 0.518 | 0.568 |
| **Perceived Behaviour Control <-> Behaviour Intention** | 0.591 | 0.757 | 0.490 | 0.562 |
| **Perceived Behaviour Control <-> Facility Availability** | 0.512 | 0.556 | 0.506 | 0.566 |
| **Perceived Behaviour Control <-> Information publicity** | 0.036 | 0.585 | 0.415 | 0.485 |
| **Situational Factor <-> Attitude** | 0.210 | 0.208 | 0.404 | 0.135 |
| **Situational Factor <-> Awareness of Benefit** | 0.162 | 0.172 | 0.328 | 0.092 |
| **Situational Factor <-> Behaviour Intention** | 0.167 | 0.243 | 0.363 | 0.117 |
| **Situational Factor <-> Facility Availability** | 0.087 | 0.107 | 0.145 | 0.100 |
| **Situational Factor <-> Information publicity** | 0.115 | 0.099 | 0.145 | 0.069 |
| **Situational Factor <-> Perceived Behaviour Control** | 0.233 | 0.236 | 0.356 | 0.272 |
| **Subjective Norm <-> Attitude** | 0.144 | 0.592 | 0.539 | 0.584 |
| **Subjective Norm <-> Awareness of Benefit** | 0.164 | 0.567 | 0.623 | 0.451 |
| **Subjective Norm <-> Behaviour Intention** | 0.232 | 0.617 | 0.598 | 0.204 |
| **Subjective Norm <-> Facility Availability** | 0.101 | 0.414 | 0.363 | 0.548 |
| **Subjective Norm <-> Information publicity** | 0.830 | 0.527 | 0.423 | 0.538 |
| **Subjective Norm <-> Perceived Behaviour Control** | 0.070 | 0.611 | 0.539 | 0.442 |
| **Subjective Norm <-> Situational Factor** | 0.159 | 0.053 | 0.270 | 0.072 |
| **Trust <-> Attitude** | 0.563 | 0.641 | 0.410 | 0.555 |
| **Trust <-> Awareness of Benefit** | 0.596 | 0.650 | 0.488 | 0.595 |
| **Trust <-> Behaviour Intention** | 0.566 | 0.716 | 0.550 | 0.425 |
| **Trust <-> Facility Availability** | 0.668 | 0.743 | 0.500 | 0.742 |
| **Trust <-> Information publicity** | 0.123 | 0.785 | 0.658 | 0.813 |
| **Trust <-> Perceived Behaviour Control** | 0.507 | 0.586 | 0.335 | 0.531 |
| **Trust <-> Situational Factor** | 0.059 | 0.065 | 0.173 | 0.083 |
| **Trust <-> Subjective Norm** | 0.095 | 0.626 | 0.494 | 0.618 |

Table B7. Result of Discriminant validity check: Confidence intervals bias corrected (Central Vietnam)

|  | **Original sample (O)** | **Sample mean (M)** | **Bias** | **2.50%** | **97.50%** |
| --- | --- | --- | --- | --- | --- |
| **Awareness of Benefit <-> Attitude** | 0.820 | 0.819 | -0.001 | 0.737 | 0.888 |
| **Behaviour Intention <-> Attitude** | 0.593 | 0.590 | -0.003 | 0.492 | 0.684 |
| **Behaviour Intention <-> Awareness of Benefit** | 0.633 | 0.632 | -0.001 | 0.53 | 0.721 |
| **Facility Availability <-> Attitude** | 0.530 | 0.527 | -0.002 | 0.423 | 0.626 |
| **Facility Availability <-> Awareness of Benefit** | 0.572 | 0.572 | 0.000 | 0.455 | 0.682 |
| **Facility Availability <-> Behaviour Intention** | 0.556 | 0.555 | -0.002 | 0.462 | 0.646 |
| **Information publicity <-> Attitude** | 0.103 | 0.103 | 0.000 | 0.045 | 0.164 |
| **Information publicity <-> Awareness of Benefit** | 0.109 | 0.109 | 0.000 | 0.041 | 0.182 |
| **Information publicity <-> Behaviour Intention** | 0.137 | 0.137 | 0.001 | 0.075 | 0.206 |
| **Information publicity <-> Facility Availability** | 0.166 | 0.167 | 0.001 | 0.092 | 0.234 |
| **Perceived Behaviour Control <-> Attitude** | 0.590 | 0.589 | -0.001 | 0.503 | 0.669 |
| **Perceived Behaviour Control <-> Awareness of Benefit** | 0.643 | 0.644 | 0.001 | 0.54 | 0.728 |
| **Perceived Behaviour Control <-> Behaviour Intention** | 0.591 | 0.590 | 0.000 | 0.503 | 0.67 |
| **Perceived Behaviour Control <-> Facility Availability** | 0.512 | 0.510 | -0.002 | 0.41 | 0.61 |
| **Perceived Behaviour Control <-> Information publicity** | 0.036 | 0.051 | 0.014 | 0.016 | 0.046 |
| **Situational Factor <-> Attitude** | 0.210 | 0.211 | 0.001 | 0.126 | 0.29 |
| **Situational Factor <-> Awareness of Benefit** | 0.162 | 0.164 | 0.001 | 0.081 | 0.253 |
| **Situational Factor <-> Behaviour Intention** | 0.167 | 0.171 | 0.003 | 0.103 | 0.238 |
| **Situational Factor <-> Facility Availability** | 0.087 | 0.094 | 0.007 | 0.05 | 0.117 |
| **Situational Factor <-> Information publicity** | 0.115 | 0.115 | 0.000 | 0.043 | 0.196 |
| **Situational Factor <-> Perceived Behaviour Control** | 0.233 | 0.233 | 0.000 | 0.153 | 0.31 |
| **Subjective Norm <-> Attitude** | 0.144 | 0.143 | -0.001 | 0.073 | 0.21 |
| **Subjective Norm <-> Awareness of Benefit** | 0.164 | 0.163 | -0.001 | 0.093 | 0.232 |
| **Subjective Norm <-> Behaviour Intention** | 0.232 | 0.231 | -0.001 | 0.16 | 0.299 |
| **Subjective Norm <-> Facility Availability** | 0.101 | 0.103 | 0.002 | 0.043 | 0.17 |
| **Subjective Norm <-> Information publicity** | 0.830 | 0.830 | 0.000 | 0.794 | 0.861 |
| **Subjective Norm <-> Perceived Behaviour Control** | 0.070 | 0.077 | 0.008 | 0.039 | 0.097 |
| **Subjective Norm <-> Situational Factor** | 0.159 | 0.159 | 0.000 | 0.084 | 0.238 |
| **Trust <-> Attitude** | 0.563 | 0.561 | -0.002 | 0.462 | 0.65 |
| **Trust <-> Awareness of Benefit** | 0.596 | 0.595 | -0.001 | 0.485 | 0.696 |
| **Trust <-> Behaviour Intention** | 0.566 | 0.564 | -0.002 | 0.471 | 0.654 |
| **Trust <-> Facility Availability** | 0.668 | 0.668 | -0.001 | 0.581 | 0.743 |
| **Trust <-> Information publicity** | 0.123 | 0.127 | 0.004 | 0.063 | 0.192 |
| **Trust <-> Perceived Behaviour Control** | 0.507 | 0.506 | -0.001 | 0.413 | 0.591 |
| **Trust <-> Situational Factor** | 0.059 | 0.076 | 0.017 | 0.025 | 0.085 |
| **Trust <-> Subjective Norm** | 0.095 | 0.099 | 0.004 | 0.053 | 0.155 |

Table B8. Result of Discriminant validity check: Confidence intervals bias corrected (Danang City – Level I))

| **Danang City (Level I)** | **Original sample (O)** | **Sample mean (M)** | **Bias** | **2.50%** | **97.50%** |
| --- | --- | --- | --- | --- | --- |
| **Awareness of Benefit <-> Attitude** | 0.854 | 0.853 | 0.000 | 0.740 | 0.939 |
| **Behaviour Intention <-> Attitude** | 0.753 | 0.746 | -0.006 | 0.630 | 0.839 |
| **Behaviour Intention <-> Awareness of Benefit** | 0.785 | 0.782 | -0.003 | 0.657 | 0.876 |
| **Facility Availability <-> Attitude** | 0.537 | 0.532 | -0.005 | 0.373 | 0.677 |
| **Facility Availability <-> Awareness of Benefit** | 0.656 | 0.656 | 0.001 | 0.468 | 0.801 |
| **Facility Availability <-> Behaviour Intention** | 0.602 | 0.600 | -0.002 | 0.450 | 0.730 |
| **Information publicity <-> Attitude** | 0.518 | 0.514 | -0.004 | 0.375 | 0.639 |
| **Information publicity <-> Awareness of Benefit** | 0.544 | 0.543 | -0.001 | 0.364 | 0.693 |
| **Information publicity <-> Behaviour Intention** | 0.471 | 0.469 | -0.002 | 0.317 | 0.600 |
| **Information publicity <-> Facility Availability** | 0.681 | 0.680 | -0.001 | 0.557 | 0.772 |
| **Perceived Behaviour Control <-> Attitude** | 0.588 | 0.583 | -0.005 | 0.449 | 0.694 |
| **Perceived Behaviour Control <-> Awareness of Benefit** | 0.773 | 0.770 | -0.004 | 0.649 | 0.869 |
| **Perceived Behaviour Control <-> Behaviour Intention** | 0.757 | 0.756 | -0.001 | 0.652 | 0.839 |
| **Perceived Behaviour Control <-> Facility Availability** | 0.556 | 0.555 | -0.002 | 0.388 | 0.690 |
| **Perceived Behaviour Control <-> Information publicity** | 0.585 | 0.583 | -0.002 | 0.445 | 0.706 |
| **Situational Factor <-> Attitude** | 0.208 | 0.210 | 0.002 | 0.078 | 0.341 |
| **Situational Factor <-> Awareness of Benefit** | 0.172 | 0.181 | 0.008 | 0.063 | 0.316 |
| **Situational Factor <-> Behaviour Intention** | 0.243 | 0.255 | 0.012 | 0.134 | 0.351 |
| **Situational Factor <-> Facility Availability** | 0.107 | 0.124 | 0.018 | 0.053 | 0.145 |
| **Situational Factor <-> Information publicity** | 0.099 | 0.117 | 0.019 | 0.032 | 0.199 |
| **Situational Factor <-> Perceived Behaviour Control** | 0.236 | 0.237 | 0.002 | 0.106 | 0.362 |
| **Subjective Norm <-> Attitude** | 0.592 | 0.586 | -0.006 | 0.450 | 0.699 |
| **Subjective Norm <-> Awareness of Benefit** | 0.567 | 0.565 | -0.002 | 0.420 | 0.687 |
| **Subjective Norm <-> Behaviour Intention** | 0.617 | 0.615 | -0.003 | 0.499 | 0.717 |
| **Subjective Norm <-> Facility Availability** | 0.414 | 0.412 | -0.001 | 0.247 | 0.559 |
| **Subjective Norm <-> Information publicity** | 0.527 | 0.527 | -0.001 | 0.389 | 0.648 |
| **Subjective Norm <-> Perceived Behaviour Control** | 0.611 | 0.61 | -0.001 | 0.500 | 0.703 |
| **Subjective Norm <-> Situational Factor** | 0.053 | 0.081 | 0.028 | 0.015 | 0.078 |
| **Trust <-> Attitude** | 0.641 | 0.635 | -0.005 | 0.486 | 0.755 |
| **Trust <-> Awareness of Benefit** | 0.650 | 0.650 | -0.001 | 0.461 | 0.796 |
| **Trust <-> Behaviour Intention** | 0.716 | 0.714 | -0.002 | 0.569 | 0.815 |
| **Trust <-> Facility Availability** | 0.743 | 0.740 | -0.003 | 0.628 | 0.834 |
| **Trust <-> Information publicity** | 0.785 | 0.783 | -0.002 | 0.691 | 0.863 |
| **Trust <-> Perceived Behaviour Control** | 0.586 | 0.583 | -0.003 | 0.433 | 0.708 |
| **Trust <-> Situational Factor** | 0.065 | 0.093 | 0.028 | 0.024 | 0.110 |
| **Trust <-> Subjective Norm** | 0.626 | 0.625 | -0.001 | 0.492 | 0.736 |

Table B9. Result of Discriminant validity check: Confidence intervals bias corrected (Hue City – Level II)

| **Hue City (Level II)** | **Original sample (O)** | **Sample mean (M)** | **Bias** | **2.50%** | **97.50%** |
| --- | --- | --- | --- | --- | --- |
| **Awareness of Benefit <-> Attitude** | 0.856 | 0.857 | 0.001 | 0.721 | 0.953 |
| **Behaviour Intention <-> Attitude** | 0.626 | 0.616 | -0.010 | 0.432 | 0.764 |
| **Behaviour Intention <-> Awareness of Benefit** | 0.637 | 0.627 | -0.009 | 0.421 | 0.782 |
| **Facility Availability <-> Attitude** | 0.557 | 0.554 | -0.003 | 0.365 | 0.701 |
| **Facility Availability <-> Awareness of Benefit** | 0.528 | 0.531 | 0.004 | 0.345 | 0.686 |
| **Facility Availability <-> Behaviour Intention** | 0.562 | 0.567 | 0.005 | 0.378 | 0.699 |
| **Information publicity <-> Attitude** | 0.455 | 0.454 | -0.002 | 0.284 | 0.599 |
| **Information publicity <-> Awareness of Benefit** | 0.504 | 0.506 | 0.002 | 0.310 | 0.657 |
| **Information publicity <-> Behaviour Intention** | 0.448 | 0.449 | 0.001 | 0.271 | 0.601 |
| **Information publicity <-> Facility Availability** | 0.621 | 0.618 | -0.003 | 0.468 | 0.750 |
| **Perceived Behaviour Control <-> Attitude** | 0.502 | 0.503 | 0.000 | 0.339 | 0.646 |
| **Perceived Behaviour Control <-> Awareness of Benefit** | 0.518 | 0.524 | 0.006 | 0.342 | 0.672 |
| **Perceived Behaviour Control <-> Behaviour Intention** | 0.490 | 0.495 | 0.005 | 0.338 | 0.615 |
| **Perceived Behaviour Control <-> Facility Availability** | 0.506 | 0.505 | -0.001 | 0.341 | 0.653 |
| **Perceived Behaviour Control <-> Information publicity** | 0.415 | 0.417 | 0.002 | 0.244 | 0.574 |
| **Situational Factor <-> Attitude** | 0.404 | 0.402 | -0.002 | 0.244 | 0.55 |
| **Situational Factor <-> Awareness of Benefit** | 0.328 | 0.329 | 0.002 | 0.151 | 0.486 |
| **Situational Factor <-> Behaviour Intention** | 0.363 | 0.363 | 0.000 | 0.213 | 0.508 |
| **Situational Factor <-> Facility Availability** | 0.145 | 0.172 | 0.026 | 0.054 | 0.284 |
| **Situational Factor <-> Information publicity** | 0.145 | 0.173 | 0.027 | 0.059 | 0.239 |
| **Situational Factor <-> Perceived Behaviour Control** | 0.356 | 0.363 | 0.007 | 0.225 | 0.485 |
| **Subjective Norm <-> Attitude** | 0.539 | 0.534 | -0.005 | 0.360 | 0.678 |
| **Subjective Norm <-> Awareness of Benefit** | 0.623 | 0.624 | 0.001 | 0.454 | 0.75 |
| **Subjective Norm <-> Behaviour Intention** | 0.598 | 0.598 | 0.000 | 0.426 | 0.72 |
| **Subjective Norm <-> Facility Availability** | 0.363 | 0.365 | 0.003 | 0.173 | 0.531 |
| **Subjective Norm <-> Information publicity** | 0.423 | 0.427 | 0.004 | 0.260 | 0.565 |
| **Subjective Norm <-> Perceived Behaviour Control** | 0.539 | 0.540 | 0.001 | 0.426 | 0.645 |
| **Subjective Norm <-> Situational Factor** | 0.270 | 0.272 | 0.002 | 0.109 | 0.442 |
| **Trust <-> Attitude** | 0.410 | 0.406 | -0.004 | 0.209 | 0.586 |
| **Trust <-> Awareness of Benefit** | 0.488 | 0.488 | 0.000 | 0.260 | 0.673 |
| **Trust <-> Behaviour Intention** | 0.550 | 0.549 | -0.001 | 0.360 | 0.702 |
| **Trust <-> Facility Availability** | 0.500 | 0.499 | -0.002 | 0.321 | 0.653 |
| **Trust <-> Information publicity** | 0.658 | 0.658 | 0.000 | 0.503 | 0.78 |
| **Trust <-> Perceived Behaviour Control** | 0.335 | 0.336 | 0.002 | 0.174 | 0.502 |
| **Trust <-> Situational Factor** | 0.173 | 0.202 | 0.029 | 0.077 | 0.259 |
| **Trust <-> Subjective Norm** | 0.494 | 0.496 | 0.002 | 0.303 | 0.642 |

Table B10. Result of Discriminant validity check: Confidence intervals bias corrected (Hoi An City – Level III)

| **Hoi An City (Level III)** | **Original sample (O)** | **Sample mean (M)** | **Bias** | **2.50%** | **97.50%** |
| --- | --- | --- | --- | --- | --- |
| **Awareness of Benefit <-> Attitude** | 0.672 | 0.675 | 0.003 | 0.436 | 0.866 |
| **Behaviour Intention <-> Attitude** | 0.406 | 0.404 | -0.002 | 0.230 | 0.585 |
| **Behaviour Intention <-> Awareness of Benefit** | 0.479 | 0.486 | 0.006 | 0.306 | 0.654 |
| **Facility Availability <-> Attitude** | 0.625 | 0.619 | -0.006 | 0.436 | 0.778 |
| **Facility Availability <-> Awareness of Benefit** | 0.542 | 0.544 | 0.002 | 0.327 | 0.749 |
| **Facility Availability <-> Behaviour Intention** | 0.496 | 0.494 | -0.002 | 0.336 | 0.644 |
| **Information publicity <-> Attitude** | 0.606 | 0.600 | -0.005 | 0.424 | 0.749 |
| **Information publicity <-> Awareness of Benefit** | 0.620 | 0.620 | 0.000 | 0.433 | 0.770 |
| **Information publicity <-> Behaviour Intention** | 0.383 | 0.381 | -0.001 | 0.228 | 0.542 |
| **Information publicity <-> Facility Availability** | 0.815 | 0.814 | -0.001 | 0.671 | 0.918 |
| **Perceived Behaviour Control <-> Attitude** | 0.728 | 0.721 | -0.006 | 0.566 | 0.850 |
| **Perceived Behaviour Control <-> Awareness of Benefit** | 0.568 | 0.575 | 0.007 | 0.334 | 0.776 |
| **Perceived Behaviour Control <-> Behaviour Intention** | 0.562 | 0.561 | -0.001 | 0.418 | 0.702 |
| **Perceived Behaviour Control <-> Facility Availability** | 0.566 | 0.556 | -0.010 | 0.363 | 0.731 |
| **Perceived Behaviour Control <-> Information publicity** | 0.485 | 0.476 | -0.009 | 0.289 | 0.651 |
| **Situational Factor <-> Attitude** | 0.135 | 0.143 | 0.008 | 0.045 | 0.266 |
| **Situational Factor <-> Awareness of Benefit** | 0.092 | 0.113 | 0.021 | 0.026 | 0.167 |
| **Situational Factor <-> Behaviour Intention** | 0.117 | 0.132 | 0.015 | 0.066 | 0.166 |
| **Situational Factor <-> Facility Availability** | 0.100 | 0.127 | 0.027 | 0.031 | 0.137 |
| **Situational Factor <-> Information publicity** | 0.069 | 0.105 | 0.036 | 0.019 | 0.104 |
| **Situational Factor <-> Perceived Behaviour Control** | 0.272 | 0.275 | 0.003 | 0.152 | 0.385 |
| **Subjective Norm <-> Attitude** | 0.584 | 0.579 | -0.005 | 0.431 | 0.716 |
| **Subjective Norm <-> Awareness of Benefit** | 0.451 | 0.454 | 0.003 | 0.278 | 0.608 |
| **Subjective Norm <-> Behaviour Intention** | 0.204 | 0.218 | 0.014 | 0.098 | 0.371 |
| **Subjective Norm <-> Facility Availability** | 0.548 | 0.548 | 0.000 | 0.362 | 0.716 |
| **Subjective Norm <-> Information publicity** | 0.538 | 0.538 | -0.001 | 0.374 | 0.684 |
| **Subjective Norm <-> Perceived Behaviour Control** | 0.442 | 0.435 | -0.007 | 0.261 | 0.612 |
| **Subjective Norm <-> Situational Factor** | 0.072 | 0.106 | 0.034 | 0.021 | 0.093 |
| **Trust <-> Attitude** | 0.555 | 0.551 | -0.004 | 0.383 | 0.695 |
| **Trust <-> Awareness of Benefit** | 0.595 | 0.597 | 0.002 | 0.432 | 0.746 |
| **Trust <-> Behaviour Intention** | 0.425 | 0.425 | 0.000 | 0.274 | 0.568 |
| **Trust <-> Facility Availability** | 0.742 | 0.742 | 0.000 | 0.572 | 0.861 |
| **Trust <-> Information publicity** | 0.813 | 0.813 | 0.001 | 0.708 | 0.889 |
| **Trust <-> Perceived Behaviour Control** | 0.531 | 0.526 | -0.005 | 0.370 | 0.663 |
| **Trust <-> Situational Factor** | 0.083 | 0.114 | 0.031 | 0.027 | 0.116 |
| **Trust <-> Subjective Norm** | 0.618 | 0.617 | -0.001 | 0.478 | 0.736 |

Table B11. Discriminant validity check: Cross loading of observed variables (Central Vietnam)

| **Item** | **Attitude** | **Awareness of Benefit** | **Behaviour Intention** | **Facility Availability** | **Information publicity** | **Perceived Behaviour Control** | **Situational Factor** | **Subjective Norm** | **Trust** |
| --- | --- | --- | --- | --- | --- | --- | --- | --- | --- |
| **AB1** | 0.541 | 0.894 | 0.47 | 0.369 | -0.089 | 0.399 | -0.143 | 0.136 | 0.372 |
| **AB2** | 0.589 | 0.858 | 0.41 | 0.413 | -0.067 | 0.486 | -0.073 | 0.096 | 0.409 |
| **AT1** | 0.907 | 0.613 | 0.476 | 0.41 | -0.083 | 0.482 | -0.166 | 0.119 | 0.438 |
| **AT2** | 0.928 | 0.592 | 0.483 | 0.405 | -0.081 | 0.504 | -0.163 | 0.109 | 0.451 |
| **AT3** | 0.873 | 0.534 | 0.472 | 0.445 | -0.106 | 0.428 | -0.155 | 0.125 | 0.397 |
| **BI1** | 0.479 | 0.48 | 0.794 | 0.443 | -0.103 | 0.456 | -0.18 | 0.173 | 0.399 |
| **BI2** | 0.48 | 0.458 | 0.82 | 0.464 | -0.114 | 0.407 | -0.176 | 0.158 | 0.417 |
| **BI3** | 0.422 | 0.388 | 0.782 | 0.371 | -0.117 | 0.403 | -0.013 | 0.136 | 0.379 |
| **BI4** | 0.396 | 0.378 | 0.78 | 0.31 | -0.098 | 0.359 | -0.077 | 0.148 | 0.357 |
| **BI5** | 0.34 | 0.309 | 0.792 | 0.359 | -0.126 | 0.409 | -0.083 | 0.225 | 0.346 |
| **BI6** | 0.388 | 0.372 | 0.805 | 0.406 | -0.077 | 0.463 | -0.142 | 0.188 | 0.393 |
| **FA1** | 0.416 | 0.417 | 0.506 | 0.851 | -0.146 | 0.402 | -0.101 | 0.093 | 0.501 |
| **FA2** | 0.284 | 0.301 | 0.307 | 0.751 | -0.127 | 0.302 | 0.021 | 0.079 | 0.38 |
| **FA3** | 0.326 | 0.287 | 0.332 | 0.776 | -0.079 | 0.33 | 0.059 | 0.03 | 0.397 |
| **FA4** | 0.451 | 0.406 | 0.422 | 0.862 | -0.118 | 0.396 | -0.054 | 0.08 | 0.501 |
| **LP1** | -0.053 | -0.072 | -0.074 | -0.141 | 0.904 | 0.005 | 0.069 | -0.681 | -0.057 |
| **LP2** | -0.112 | -0.095 | -0.124 | -0.146 | 0.952 | -0.012 | 0.097 | -0.721 | -0.103 |
| **LP3** | -0.098 | -0.082 | -0.149 | -0.13 | 0.946 | -0.009 | 0.114 | -0.774 | -0.135 |
| **PBC1** | 0.448 | 0.446 | 0.448 | 0.394 | -0.043 | 0.801 | -0.138 | 0.098 | 0.382 |
| **PBC2** | 0.405 | 0.386 | 0.397 | 0.319 | 0.013 | 0.785 | -0.169 | 0.012 | 0.338 |
| **PBC3** | 0.407 | 0.421 | 0.425 | 0.365 | -0.023 | 0.809 | -0.138 | 0.079 | 0.339 |
| **PBC4** | 0.449 | 0.409 | 0.435 | 0.378 | 0.037 | 0.885 | -0.182 | -0.015 | 0.349 |
| **PBC5** | 0.446 | 0.405 | 0.459 | 0.381 | -0.012 | 0.844 | -0.18 | 0.024 | 0.367 |
| **SF1** | -0.158 | -0.106 | -0.116 | -0.023 | 0.099 | -0.192 | 0.894 | -0.149 | 0.003 |
| **SF2** | -0.169 | -0.123 | -0.149 | -0.043 | 0.093 | -0.17 | 0.938 | -0.108 | 0.048 |
| **SN1** | 0.1 | 0.106 | 0.216 | 0.101 | -0.763 | 0.024 | -0.14 | 0.938 | 0.042 |
| **SN2** | 0.14 | 0.141 | 0.221 | 0.09 | -0.705 | 0.078 | -0.134 | 0.954 | 0.103 |
| **SN3** | 0.131 | 0.132 | 0.16 | 0.055 | -0.75 | 0.03 | -0.109 | 0.934 | 0.069 |
| **TRU1** | 0.39 | 0.4 | 0.374 | 0.48 | -0.125 | 0.308 | 0.045 | 0.068 | 0.792 |
| **TRU2** | 0.368 | 0.375 | 0.379 | 0.451 | -0.071 | 0.301 | 0.011 | 0.042 | 0.766 |
| **TRU3** | 0.399 | 0.34 | 0.356 | 0.393 | -0.063 | 0.335 | 0.069 | 0.002 | 0.814 |
| **TRU4** | 0.357 | 0.301 | 0.417 | 0.441 | -0.102 | 0.415 | -0.021 | 0.119 | 0.806 |

*Note: Food waste separation intention (BI), Attitude (AT), Awareness of Benefit (AB), Information Publicity (IP), Situational Factor (SF), Facility Availability (FA), Trust (Tru), Perceived Behaviour Control (PBC), and Subjective Norm (SN)*

Table B12. Discriminant validity check: Cross loading of observed variables (Danang City – Level I)

| **Item** | **Attitude** | **Awareness of Benefit** | **Behaviour Intention** | **Facility Availability** | **Information publicity** | **Perceived Behaviour Control** | **Situational Factor** | **Subjective Norm** | **Trust** |
| --- | --- | --- | --- | --- | --- | --- | --- | --- | --- |
| **AB1** | 0.556 | 0.879 | 0.545 | 0.423 | -0.305 | 0.48 | -0.157 | 0.356 | 0.412 |
| **AB2** | 0.627 | 0.866 | 0.519 | 0.467 | -0.418 | 0.554 | -0.068 | 0.4 | 0.439 |
| **AT1** | 0.939 | 0.644 | 0.648 | 0.472 | -0.456 | 0.494 | -0.169 | 0.497 | 0.528 |
| **AT2** | 0.933 | 0.631 | 0.61 | 0.423 | -0.439 | 0.507 | -0.145 | 0.476 | 0.513 |
| **AT3** | 0.91 | 0.608 | 0.617 | 0.462 | -0.419 | 0.456 | -0.186 | 0.478 | 0.504 |
| **BI1** | 0.573 | 0.552 | 0.825 | 0.462 | -0.349 | 0.578 | -0.215 | 0.463 | 0.513 |
| **BI2** | 0.586 | 0.519 | 0.823 | 0.458 | -0.327 | 0.499 | -0.229 | 0.37 | 0.489 |
| **BI3** | 0.546 | 0.472 | 0.722 | 0.391 | -0.411 | 0.477 | 0.008 | 0.365 | 0.463 |
| **BI4** | 0.553 | 0.441 | 0.741 | 0.352 | -0.313 | 0.456 | -0.126 | 0.349 | 0.453 |
| **BI5** | 0.45 | 0.43 | 0.788 | 0.433 | -0.321 | 0.531 | -0.165 | 0.469 | 0.468 |
| **BI6** | 0.474 | 0.455 | 0.809 | 0.438 | -0.304 | 0.56 | -0.222 | 0.519 | 0.469 |
| **FA1** | 0.434 | 0.452 | 0.542 | 0.873 | -0.504 | 0.462 | -0.088 | 0.288 | 0.563 |
| **FA2** | 0.325 | 0.384 | 0.339 | 0.778 | -0.408 | 0.353 | 0.085 | 0.281 | 0.431 |
| **FA3** | 0.36 | 0.392 | 0.388 | 0.82 | -0.509 | 0.378 | 0.123 | 0.316 | 0.531 |
| **FA4** | 0.496 | 0.474 | 0.502 | 0.895 | -0.549 | 0.433 | -0.001 | 0.328 | 0.589 |
| **LP1** | -0.296 | -0.325 | -0.231 | -0.474 | 0.82 | -0.424 | -0.117 | -0.285 | -0.472 |
| **LP2** | -0.451 | -0.411 | -0.361 | -0.527 | 0.898 | -0.45 | -0.073 | -0.36 | -0.579 |
| **LP3** | -0.436 | -0.333 | -0.45 | -0.516 | 0.868 | -0.424 | -0.022 | -0.5 | -0.631 |
| **PBC1** | 0.393 | 0.522 | 0.489 | 0.374 | -0.384 | 0.76 | -0.141 | 0.431 | 0.427 |
| **PBC2** | 0.433 | 0.435 | 0.511 | 0.338 | -0.341 | 0.76 | -0.178 | 0.395 | 0.38 |
| **PBC3** | 0.368 | 0.502 | 0.516 | 0.398 | -0.383 | 0.769 | -0.132 | 0.381 | 0.374 |
| **PBC4** | 0.451 | 0.458 | 0.568 | 0.416 | -0.437 | 0.878 | -0.187 | 0.432 | 0.389 |
| **PBC5** | 0.46 | 0.474 | 0.567 | 0.435 | -0.457 | 0.852 | -0.155 | 0.476 | 0.416 |
| **SF1** | -0.163 | -0.13 | -0.191 | 0.032 | -0.081 | -0.166 | 0.918 | -0.013 | 0.03 |
| **SF2** | -0.166 | -0.107 | -0.189 | 0.002 | -0.047 | -0.197 | 0.916 | 0.037 | 0.049 |
| **SN1** | 0.47 | 0.417 | 0.524 | 0.354 | -0.384 | 0.494 | -0.027 | 0.861 | 0.417 |
| **SN2** | 0.447 | 0.348 | 0.483 | 0.321 | -0.406 | 0.455 | 0.024 | 0.919 | 0.491 |
| **SN3** | 0.448 | 0.364 | 0.386 | 0.247 | -0.439 | 0.417 | 0.048 | 0.835 | 0.453 |
| **TRU1** | 0.503 | 0.451 | 0.511 | 0.629 | -0.578 | 0.404 | 0.035 | 0.399 | 0.827 |
| **TRU2** | 0.413 | 0.403 | 0.481 | 0.502 | -0.431 | 0.352 | 0.052 | 0.332 | 0.763 |
| **TRU3** | 0.417 | 0.332 | 0.432 | 0.428 | -0.6 | 0.34 | 0.07 | 0.417 | 0.797 |
| **TRU4** | 0.441 | 0.367 | 0.509 | 0.458 | -0.529 | 0.47 | -0.013 | 0.508 | 0.814 |

*Note: Food waste separation intention (BI), Attitude (AT), Awareness of Benefit (AB), Information Publicity (IP), Situational Factor (SF), Facility Availability (FA), Trust (Tru), Perceived Behaviour Control (PBC), and Subjective Norm (SN)*

Table B13. Discriminant validity check: Cross loading of observed variables (Hue City – Level II)

| **Item** | **Attitude** | **Awareness of Benefit** | **Behaviour Intention** | **Facility Availability** | **Information publicity** | **Perceived Behaviour Control** | **Situational Factor** | **Subjective Norm** | **Trust** |
| --- | --- | --- | --- | --- | --- | --- | --- | --- | --- |
| **AB1** | 0.661 | 0.895 | 0.46 | 0.283 | -0.338 | 0.347 | -0.282 | 0.447 | 0.309 |
| **AB2** | 0.617 | 0.908 | 0.491 | 0.477 | -0.379 | 0.444 | -0.173 | 0.468 | 0.378 |
| **AT1** | 0.93 | 0.677 | 0.505 | 0.397 | -0.348 | 0.481 | -0.349 | 0.47 | 0.294 |
| **AT2** | 0.949 | 0.684 | 0.551 | 0.433 | -0.391 | 0.454 | -0.357 | 0.476 | 0.376 |
| **AT3** | 0.821 | 0.546 | 0.446 | 0.415 | -0.302 | 0.304 | -0.195 | 0.331 | 0.264 |
| **BI1** | 0.514 | 0.528 | 0.751 | 0.472 | -0.251 | 0.351 | -0.263 | 0.389 | 0.328 |
| **BI2** | 0.512 | 0.5 | 0.777 | 0.512 | -0.292 | 0.337 | -0.282 | 0.368 | 0.378 |
| **BI3** | 0.325 | 0.361 | 0.746 | 0.335 | -0.304 | 0.297 | -0.092 | 0.348 | 0.326 |
| **BI4** | 0.375 | 0.337 | 0.735 | 0.225 | -0.254 | 0.251 | -0.29 | 0.34 | 0.324 |
| **BI5** | 0.38 | 0.308 | 0.812 | 0.348 | -0.306 | 0.361 | -0.216 | 0.51 | 0.395 |
| **BI6** | 0.418 | 0.36 | 0.784 | 0.354 | -0.354 | 0.439 | -0.222 | 0.492 | 0.426 |
| **FA1** | 0.339 | 0.381 | 0.513 | 0.81 | -0.346 | 0.321 | -0.169 | 0.247 | 0.399 |
| **FA2** | 0.286 | 0.292 | 0.332 | 0.731 | -0.407 | 0.288 | -0.042 | 0.145 | 0.367 |
| **FA3** | 0.412 | 0.28 | 0.32 | 0.772 | -0.357 | 0.363 | -0.047 | 0.27 | 0.177 |
| **FA4** | 0.415 | 0.345 | 0.327 | 0.799 | -0.361 | 0.398 | -0.099 | 0.298 | 0.335 |
| **LP1** | -0.25 | -0.269 | -0.213 | -0.455 | 0.737 | -0.217 | -0.022 | -0.164 | -0.32 |
| **LP2** | -0.348 | -0.347 | -0.328 | -0.416 | 0.886 | -0.308 | 0.116 | -0.267 | -0.44 |
| **LP3** | -0.346 | -0.36 | -0.375 | -0.334 | 0.854 | -0.361 | 0.148 | -0.425 | -0.513 |
| **PBC1** | 0.421 | 0.375 | 0.398 | 0.399 | -0.323 | 0.859 | -0.179 | 0.428 | 0.298 |
| **PBC2** | 0.347 | 0.395 | 0.351 | 0.305 | -0.332 | 0.84 | -0.264 | 0.35 | 0.193 |
| **PBC3** | 0.339 | 0.339 | 0.326 | 0.323 | -0.232 | 0.838 | -0.174 | 0.465 | 0.259 |
| **PBC4** | 0.438 | 0.392 | 0.418 | 0.409 | -0.35 | 0.934 | -0.348 | 0.438 | 0.251 |
| **PBC5** | 0.46 | 0.421 | 0.448 | 0.445 | -0.36 | 0.905 | -0.378 | 0.431 | 0.303 |
| **SF1** | -0.321 | -0.225 | -0.286 | -0.123 | 0.142 | -0.388 | 0.912 | -0.253 | -0.205 |
| **SF2** | -0.288 | -0.226 | -0.256 | -0.104 | 0.062 | -0.166 | 0.889 | -0.147 | 0.005 |
| **SN1** | 0.432 | 0.436 | 0.563 | 0.321 | -0.291 | 0.432 | -0.209 | 0.881 | 0.387 |
| **SN2** | 0.362 | 0.434 | 0.415 | 0.23 | -0.353 | 0.389 | -0.18 | 0.916 | 0.394 |
| **SN3** | 0.465 | 0.48 | 0.404 | 0.246 | -0.341 | 0.452 | -0.204 | 0.854 | 0.339 |
| **TRU1** | 0.262 | 0.323 | 0.31 | 0.338 | -0.483 | 0.208 | -0.065 | 0.277 | 0.777 |
| **TRU2** | 0.288 | 0.35 | 0.389 | 0.341 | -0.361 | 0.179 | -0.141 | 0.245 | 0.784 |
| **TRU3** | 0.303 | 0.314 | 0.325 | 0.254 | -0.524 | 0.218 | -0.078 | 0.355 | 0.818 |
| **TRU4** | 0.262 | 0.248 | 0.456 | 0.39 | -0.356 | 0.326 | -0.084 | 0.449 | 0.809 |

*Note: Food waste separation intention (BI), Attitude (AT), Awareness of Benefit (AB), Information Publicity (IP), Situational Factor (SF), Facility Availability (FA), Trust (Tru), Perceived Behaviour Control (PBC), and Subjective Norm (SN)*

Table B14. Discriminant validity check: Cross loading of observed variables (Hoi An City – Level III)

| **Item** | **Attitude** | **Awareness of Benefit** | **Behaviour Intention** | **Facility Availability** | **Information publicity** | **Perceived Behaviour Control** | **Situational Factor** | **Subjective Norm** | **Trust** |
| --- | --- | --- | --- | --- | --- | --- | --- | --- | --- |
| **AB1** | 0.384 | 0.923 | 0.393 | 0.365 | 0.354 | 0.345 | 0.035 | -0.278 | 0.374 |
| **AB2** | 0.445 | 0.773 | 0.239 | 0.339 | 0.417 | 0.38 | -0.02 | -0.3 | 0.352 |
| **AT1** | 0.806 | 0.459 | 0.284 | 0.429 | 0.432 | 0.446 | -0.041 | -0.413 | 0.429 |
| **AT2** | 0.883 | 0.36 | 0.301 | 0.449 | 0.462 | 0.569 | -0.054 | -0.448 | 0.385 |
| **AT3** | 0.88 | 0.395 | 0.321 | 0.471 | 0.394 | 0.565 | -0.072 | -0.41 | 0.339 |
| **BI1** | 0.338 | 0.355 | 0.769 | 0.387 | 0.302 | 0.431 | 0.037 | -0.243 | 0.31 |
| **BI2** | 0.358 | 0.388 | 0.838 | 0.44 | 0.359 | 0.432 | 0.079 | -0.257 | 0.405 |
| **BI3** | 0.358 | 0.322 | 0.874 | 0.374 | 0.279 | 0.461 | 0.101 | -0.151 | 0.333 |
| **BI4** | 0.216 | 0.353 | 0.871 | 0.331 | 0.214 | 0.393 | 0.179 | -0.069 | 0.276 |
| **BI5** | 0.194 | 0.188 | 0.789 | 0.274 | 0.173 | 0.361 | 0.159 | -0.031 | 0.181 |
| **BI6** | 0.273 | 0.292 | 0.816 | 0.392 | 0.334 | 0.39 | 0.098 | -0.168 | 0.285 |
| **FA1** | 0.505 | 0.416 | 0.459 | 0.865 | 0.56 | 0.467 | -0.007 | -0.393 | 0.543 |
| **FA2** | 0.306 | 0.224 | 0.237 | 0.706 | 0.509 | 0.318 | 0.095 | -0.321 | 0.392 |
| **FA3** | 0.313 | 0.193 | 0.264 | 0.701 | 0.471 | 0.278 | -0.012 | -0.391 | 0.389 |
| **FA4** | 0.464 | 0.382 | 0.37 | 0.849 | 0.528 | 0.406 | -0.069 | -0.325 | 0.52 |
| **LP1** | 0.356 | 0.301 | 0.235 | 0.516 | 0.815 | 0.304 | 0.016 | -0.358 | 0.506 |
| **LP2** | 0.42 | 0.425 | 0.273 | 0.556 | 0.863 | 0.356 | -0.007 | -0.439 | 0.591 |
| **LP3** | 0.48 | 0.379 | 0.335 | 0.592 | 0.871 | 0.378 | 0.013 | -0.327 | 0.565 |
| **PBC1** | 0.585 | 0.393 | 0.448 | 0.458 | 0.423 | 0.805 | -0.074 | -0.291 | 0.38 |
| **PBC2** | 0.389 | 0.267 | 0.327 | 0.355 | 0.323 | 0.745 | -0.174 | -0.32 | 0.364 |
| **PBC3** | 0.572 | 0.359 | 0.42 | 0.396 | 0.349 | 0.818 | -0.153 | -0.283 | 0.327 |
| **PBC4** | 0.449 | 0.338 | 0.35 | 0.377 | 0.289 | 0.83 | -0.088 | -0.3 | 0.327 |
| **PBC5** | 0.387 | 0.246 | 0.388 | 0.294 | 0.208 | 0.723 | -0.081 | -0.32 | 0.304 |
| **SF1** | -0.076 | 0.005 | 0.118 | -0.014 | 0.018 | -0.162 | 0.996 | -0.029 | 0.071 |
| **SF2** | -0.13 | -0.086 | -0.014 | -0.05 | 0.077 | -0.257 | 0.638 | -0.029 | 0.024 |
| **SN1** | -0.471 | -0.332 | -0.18 | -0.421 | -0.382 | -0.338 | 0.016 | 0.878 | -0.435 |
| **SN2** | -0.35 | -0.233 | -0.108 | -0.342 | -0.4 | -0.246 | -0.116 | 0.826 | -0.461 |
| **SN3** | -0.441 | -0.28 | -0.183 | -0.4 | -0.369 | -0.385 | -0.01 | 0.897 | -0.421 |
| **TRU1** | 0.277 | 0.372 | 0.287 | 0.405 | 0.461 | 0.231 | 0.113 | -0.268 | 0.744 |
| **TRU2** | 0.341 | 0.331 | 0.256 | 0.468 | 0.506 | 0.319 | 0.028 | -0.348 | 0.74 |
| **TRU3** | 0.446 | 0.362 | 0.32 | 0.503 | 0.586 | 0.391 | 0.107 | -0.468 | 0.841 |
| **TRU4** | 0.319 | 0.248 | 0.272 | 0.5 | 0.482 | 0.415 | -0.028 | -0.468 | 0.797 |

*Note: Food waste separation intention (BI), Attitude (AT), Awareness of Benefit (AB), Information Publicity (IP), Situational Factor (SF), Facility Availability (FA), Trust (Tru), Perceived Behaviour Control (PBC), and Subjective Norm (SN)*

# C. Inner model analysis

Table C1. Result of Multicollinearity check (VIF)

|  | **Central Vietnam** | **Danang City (Level I)** | **Hue City (Level II)** | **Hoi An City (Level III)** |
| --- | --- | --- | --- | --- |
| **Attitude -> Behaviour Intention** | 2.028 | 2.351 | 2.338 | 2.086 |
| **Awareness of Benefit -> Behaviour Intention** | 1.898 | 2.237 | 2.245 | 1.44 |
| **Facility Availability -> Behaviour Intention** | 1.674 | 2.016 | 1.593 | 2.137 |
| **Information Publicity -> Behaviour Intention** | 2.677 | 2.114 | 1.619 | 2.26 |
| **Perceived Behaviour Control -> Behaviour Intention** | 1.637 | 2.051 | 1.619 | 1.815 |
| **Situational Factor -> Behaviour Intention** | 1.099 | 1.15 | 1.184 | 1.049 |
| **Subjective Norm -> Behaviour Intention** | 2.659 | 1.729 | 1.653 | 1.54 |
| **Trust -> Behaviour Intention** | 1.682 | 2.452 | 1.587 | 2.138 |

Table C2. Result from Bootstrapping analysis for structure model (Central Vietnam)

| **Central Vietnam** | **Original sample (O)** | **Sample mean (M)** | **Standard deviation (STDEV)** | **T statistics (\|O/STDEV\|)** | **P values** |
| --- | --- | --- | --- | --- | --- |
| **Attitude -> Behaviour Intention** | 0.151 | 0.147 | 0.050 | 3.015 | 0.003 |
| **Awareness of Benefit -> Behaviour Intention** | 0.121 | 0.122 | 0.050 | 2.433 | 0.015 |
| **Facility Availability -> Behaviour Intention** | 0.186 | 0.187 | 0.037 | 4.969 | 0.000 |
| **Information publicity -> Behaviour Intention** | 0.129 | 0.129 | 0.043 | 3.039 | 0.002 |
| **Perceived Behaviour Control -> Behaviour Intention** | 0.217 | 0.218 | 0.039 | 5.505 | 0.000 |
| **Situational Factor -> Behaviour Intention** | -0.041 | -0.041 | 0.023 | 1.770 | 0.077 |
| **Subjective Norm -> Behaviour Intention** | 0.236 | 0.236 | 0.046 | 5.085 | 0.000 |
| **Trust -> Behaviour Intention** | 0.158 | 0.158 | 0.037 | 4.290 | 0.000 |

Table C3. Result from Bootstrapping analysis for structure model (Danang City – Level I)

| **Danang (Level I)** | **Original sample (O)** | **Sample mean (M)** | **Standard deviation (STDEV)** | **T statistics (\|O/STDEV\|)** | **P values** |
| --- | --- | --- | --- | --- | --- |
| **Attitude -> Behaviour Intention** | 0.272 | 0.263 | 0.07 | 3.894 | 0 |
| **Awareness of Benefit -> Behaviour Intention** | 0.073 | 0.073 | 0.062 | 1.172 | 0.241 |
| **Facility Availability -> Behaviour Intention** | 0.126 | 0.131 | 0.056 | 2.235 | 0.025 |
| **Information publicity -> Behaviour Intention** | 0.165 | 0.162 | 0.052 | 3.138 | 0.002 |
| **Perceived Behaviour Control -> Behaviour Intention** | 0.292 | 0.292 | 0.056 | 5.19 | 0 |
| **Situational Factor -> Behaviour Intention** | -0.094 | -0.096 | 0.032 | 2.911 | 0.004 |
| **Subjective Norm -> Behaviour Intention** | 0.117 | 0.118 | 0.042 | 2.744 | 0.006 |
| **Trust -> Behaviour Intention** | 0.249 | 0.249 | 0.057 | 4.33 | 0 |

Table C4. Result from Bootstrapping analysis for structure model (Hue City – Level II)

| **Hue – Level II** | **Original sample (O)** | **Sample mean (M)** | **Standard deviation (STDEV)** | **T statistics (\|O/STDEV\|)** | **P values** |
| --- | --- | --- | --- | --- | --- |
| **Attitude -> Behaviour Intention** | 0.176 | 0.168 | 0.087 | 2.028 | 0.043 |
| **Awareness of Benefit -> Behaviour Intention** | 0.094 | 0.081 | 0.108 | 0.866 | 0.387 |
| **Facility Availability -> Behaviour Intention** | 0.214 | 0.227 | 0.070 | 3.059 | 0.002 |
| **Information publicity -> Behaviour Intention** | 0.031 | 0.039 | 0.069 | 0.455 | 0.649 |
| **Perceived Behaviour Control -> Behaviour Intention** | 0.047 | 0.057 | 0.073 | 0.640 | 0.522 |
| **Situational Factor -> Behaviour Intention** | -0.108 | -0.105 | 0.054 | 2.004 | 0.045 |
| **Subjective Norm -> Behaviour Intention** | 0.225 | 0.226 | 0.073 | 3.071 | 0.002 |
| **Trust -> Behaviour Intention** | 0.182 | 0.188 | 0.058 | 3.125 | 0.002 |

Table C5. Result from Bootstrapping analysis for structure model (Hoi An City – Level III)

| **Hoi An (Level III)** | **Original sample (O)** | **Sample mean (M)** | **Standard deviation (STDEV)** | **T statistics (\|O/STDEV\|)** | **P values** |
| --- | --- | --- | --- | --- | --- |
| **Attitude -> Behaviour Intention** | -0.031 | -0.037 | 0.073 | 0.433 | 0.665 |
| **Awareness of Benefit -> Behaviour Intention** | 0.165 | 0.173 | 0.083 | 1.990 | 0.047 |
| **Facility Availability -> Behaviour Intention** | 0.248 | 0.246 | 0.077 | 3.224 | 0.001 |
| **Information publicity -> Behaviour Intention** | -0.025 | -0.025 | 0.091 | 0.281 | 0.779 |
| **Perceived Behaviour Control -> Behaviour Intention** | 0.393 | 0.386 | 0.074 | 5.339 | 0.000 |
| **Situational Factor -> Behaviour Intention** | 0.184 | 0.156 | 0.081 | 2.259 | 0.024 |
| **Subjective Norm -> Behaviour Intention** | 0.139 | 0.135 | 0.058 | 2.378 | 0.017 |
| **Trust -> Behaviour Intention** | 0.063 | 0.065 | 0.069 | 0.910 | 0.363 |

# D. Procedure of Measurement invariance of composite models (MICOM)

Table D1. MICOM Step 3: Equal mean and equal variance (Danang city – Level I and Hoi An city – Level III)

| **Danang (Level I) vs Hoi An (Level III)** | | | | | | | | | | | | | |
| --- | --- | --- | --- | --- | --- | --- | --- | --- | --- | --- | --- | --- | --- |
|  | **Original difference** | **Permutation mean difference** | **0.50%** | **99.50%** | **Permutation p value** | **Equal Mean Value?** | **Original difference** | **Permutation mean difference** | **0.50%** | **99.50%** | **Permutation p value** | **Equal Variances?** | **Full measurement Invariance?** |
| **Attitude** | -0.018 | -0.001 | -0.197 | 0.196 | 0.802 | Yes | 0.53 | 0.009 | -0.671 | 0.738 | 0.053 | Yes | Yes |
| **Awareness of Benefit** | -0.034 | -0.001 | -0.193 | 0.191 | 0.642 | Yes | 0.298 | 0.008 | -0.596 | 0.614 | 0.212 | Yes | Yes |
| **Behaviour Intention** | 0.006 | -0.001 | -0.198 | 0.189 | 0.942 | Yes | -0.155 | 0.005 | -0.501 | 0.546 | 0.447 | Yes | Yes |
| **Facility Availability** | -0.036 | 0 | -0.188 | 0.189 | 0.652 | Yes | 0.304 | 0.005 | -0.559 | 0.569 | 0.167 | Yes | Yes |
| **Information Publicity** | -1.689 | 0 | -0.196 | 0.189 | 0 | No | 0.458 | 0 | -0.13 | 0.133 | 0 | No | No |
| **Perceived Behaviour Control** | -0.394 | -0.001 | -0.197 | 0.195 | 0 | No | 0.483 | 0.007 | -0.484 | 0.525 | 0.013 | No | No |
| **Situational Factor** | -0.187 | 0.001 | -0.199 | 0.182 | 0.01 | No | 0.043 | 0.001 | -0.165 | 0.176 | 0.514 | Yes | No |
| **Subjective Norm** | 1.636 | 0.002 | -0.189 | 0.2 | 0 | No | 0.351 | 0.002 | -0.138 | 0.149 | 0 | No | No |
| **Trust** | -0.148 | -0.001 | -0.195 | 0.192 | 0.047 | No | 0.393 | 0.003 | -0.428 | 0.423 | 0.017 | No | No |

Table D2. MICOM Step 3: Equal mean and equal variance (Danang city – Level I and Hue city – Level II)

| **Danang (Level I) vs Hue (Level II)** | | | | | | | | | | | | | |
| --- | --- | --- | --- | --- | --- | --- | --- | --- | --- | --- | --- | --- | --- |
|  | **Original difference** | **Permutation mean difference** | **0.50%** | **99.50%** | **Permutation p value** | **Equal Mean Value?** | **Original difference** | **Permutation mean difference** | **0.50%** | **99.50%** | **Permutation p value** | **Equal Variances?** | **Full measurement Invariance?** |
| **Attitude** | 0.284 | -0.001 | -0.198 | 0.216 | 0 | No | 0.029 | 0.01 | -0.607 | 0.66 | 0.911 | Yes | No |
| **Awareness of Benefit** | 0.176 | -0.001 | -0.196 | 0.203 | 0.023 | No | -0.05 | 0.013 | -0.617 | 0.657 | 0.839 | Yes | No |
| **Behaviour Intention** | -0.134 | -0.002 | -0.198 | 0.205 | 0.084 | Yes | 0.248 | 0.012 | -0.554 | 0.591 | 0.271 | Yes | Yes |
| **Facility Availability** | -0.203 | 0 | -0.21 | 0.199 | 0.011 | No | 0.714 | 0.007 | -0.583 | 0.619 | 0.002 | No | No |
| **Information publicity** | -0.273 | 0 | -0.195 | 0.197 | 0 | No | -0.117 | 0.005 | -0.363 | 0.398 | 0.423 | Yes | No |
| **Perceived Behaviour Control** | 0.074 | -0.001 | -0.209 | 0.2 | 0.347 | Yes | -0.187 | 0.009 | -0.427 | 0.461 | 0.276 | Yes | Yes |
| **Situational Factor** | 0.229 | 0.002 | -0.197 | 0.2 | 0.003 | No | 0.226 | 0.004 | -0.195 | 0.215 | 0.005 | No | No |
| **Subjective Norm** | 0.145 | -0.001 | -0.203 | 0.206 | 0.061 | Yes | -0.1 | 0.005 | -0.368 | 0.406 | 0.51 | Yes | Yes |
| **Trust** | 0.193 | 0 | -0.208 | 0.204 | 0.015 | No | 0.149 | 0.003 | -0.423 | 0.45 | 0.383 | Yes | No |

Table D3. MICOM Step 3: Equal mean and equal variance (Hue city – Level II and Hoi An city – Level III)

| **Hue (Level II) vs Hoi An (Level III)** | | | | | | | | | | | | | |
| --- | --- | --- | --- | --- | --- | --- | --- | --- | --- | --- | --- | --- | --- |
|  | **Original difference** | **Permutation mean difference** | **0.50%** | **99.50%** | **Permutation p value** | **Equal Mean Value?** | **Original difference** | **Permutation mean difference** | **0.50%** | **99.50%** | **Permutation p value** | **Equal Variances?** | **Full measurement Invariance?** |
| **Attitude** | -0.338 | 0.001 | -0.213 | 0.225 | 0 | No | 0.489 | -0.005 | -0.657 | 0.611 | 0.052 | Yes | No |
| **Awareness of Benefit** | -0.212 | 0.001 | -0.217 | 0.218 | 0.014 | No | 0.335 | -0.004 | -0.667 | 0.625 | 0.196 | Yes | No |
| **Behaviour Intention** | 0.14 | 0.002 | -0.218 | 0.206 | 0.1 | Yes | -0.405 | -0.008 | -0.595 | 0.565 | 0.078 | Yes | Yes |
| **Facility Availability** | 0.182 | 0.001 | -0.219 | 0.21 | 0.034 | No | -0.389 | -0.004 | -0.575 | 0.572 | 0.087 | Yes | No |
| **Information publicity** | -1.618 | 0 | -0.227 | 0.236 | 0 | No | 0.564 | 0 | -0.176 | 0.169 | 0 | No | No |
| **Perceived Behaviour Control** | -0.449 | 0.001 | -0.215 | 0.216 | 0 | No | 0.661 | -0.004 | -0.556 | 0.54 | 0.002 | No | No |
| **Situational Factor** | -0.41 | 0.001 | -0.208 | 0.215 | 0 | No | -0.174 | -0.001 | -0.216 | 0.195 | 0.025 | No | No |
| **Subjective Norm** | 1.592 | 0 | -0.221 | 0.227 | 0 | No | 0.47 | -0.001 | -0.175 | 0.179 | 0 | No | No |
| **Trust** | -0.365 | 0 | -0.211 | 0.214 | 0 | No | 0.255 | 0 | -0.421 | 0.402 | 0.124 | Yes | No |

# Reference

Adu-Gyamfi G, Asamoah AN, Nketiah E, Obuobi B, Adjei M, Cudjoe D, Zhu B (2023) Reducing waste management challenges: Empirical assessment of waste sorting intention among corporate employees in Ghana. J Retail Consum Serv 72. https://doi.org/10.1016/j.jretconser.2023.103261

Cheah J-H, Amaro S, Roldán JL (2023) Multigroup analysis of more than two groups in PLS-SEM: A review, illustration, and recommendations. J Bus Res 156. https://doi.org/10.1016/j.jbusres.2022.113539

Chun T'ing L, Moorthy K, Gunasaygaran N, Sek Li C, Omapathi D, Jia Yi H, Anandan K, Sivakumar K (2021) Intention to reduce food waste: A study among Malaysians. J Air Waste Manag Assoc 71(7): 890-905. https://doi.org/10.1080/10962247.2021.1900001

Cohen J (1992) A power primer. Psychol Bull 112(1): 155–159. https://doi.org/10.1037/0033-2909.112.1.155

Giang HM, Takeshi F, Song Toan PP (2017) Municipal waste generation and composition in a tourist city - Hoi An, Vietnam. Journal of Japan Society of Civil Engineers 5(1): 123-132.

GSOV (2022) General Statistical Data. Hanoi, Vietnam (In Vietnamese).

Hair JF, G. Tomas MH, Christian MR, Marko S (2017) A Primer on Partial Least Squares Structural Equation Modeling (PLS-SEM), 2nd ed. SAGE Publications, Inc.

Hair JF, Risher JJ, Sarstedt M, Ringle CM (2019) When to use and how to report the results of PLS-SEM. Eur Bus Rev 31(1): 2-24. https://doi.org/10.1108/ebr-11-2018-0203

Henseler J, R. Sinkovics R-JBJ, Daekwan Kim R, Ringle CM, Sarstedt M (2016) Testing measurement invariance of composites using partial least squares. Int Mark Rev 33(3): 405-431. https://doi.org/10.1108/imr-09-2014-0304

Karim Ghani WA, Rusli IF, Biak DR, Idris A (2013) An application of the theory of planned behaviour to study the influencing factors of participation in source separation of food waste. Waste Manage 33(5): 1276-1281. https://doi.org/10.1016/j.wasman.2012.09.019

Kock N (2015) Common Method Bias in PLS-SEM. Int J e-Collab 11(4): 1-10. https://doi.org/10.4018/ijec.2015100101

Kock N, Lynn G (2012) Lateral Collinearity and Misleading Results in Variance-Based SEM: An Illustration and Recommendations. J Assoc Inf Syst 13(7): 546-580. https://doi.org/10.17705/1jais.00302

Krejcie RV, Morgan DW (1970) Determining Sample Size for Research Activities. Educational and Psychological Measurement 30(3): 607-610. https://doi.org/10.1177/001316447003000308

Li Y, Bhutto MY, Sun C, Mehdi SM (2023) Do information publicity and moral norms trigger waste-sorting intention among households? A sequential mediation analysis. Front Psychol 14: 1193411. https://doi.org/10.3389/fpsyg.2023.1193411

Loan LTT, Nomura H, Takahashi Y, Yabe M (2017) Psychological driving forces behind households’ behaviors toward municipal organic waste separation at source in Vietnam: a structural equation modeling approach. J Mater Cycles Waste Manag 19(3): 1052-1060. https://doi.org/10.1007/s10163-017-0587-3

MONRE (2021) Báo cáo Hiện trạng Môi trường Quốc gia. Hanoi, Vietnam (In Vietnamese).

Ng PY, Ho P-L, Sia JK-M (2021) Integrative model of behavioural intention: the influence of environmental concern and condition factors on food waste separation. Manag Environ Qual 32(3): 631-645. https://doi.org/10.1108/meq-06-2020-0128

Nhung NTK (2023) The determinants of individuals’ waste separation intention in an urbanizing city: A case study of Hanoi, Vietnam. Habitat Int 137: 102835. https://doi.org/10.1016/j.habitatint.2023.102835

Oehman JM, Babbitt CW, Flynn C (2022) What predicts and prevents source separation of household food waste? An application of the theory of planned behavior. Resour Conserv Recycl 186: 106492. https://doi.org/10.1016/j.resconrec.2022.106492

PCDC (2023) Plan No. 103/KH-UBND dated May 10, 2023 on implementation of solid waste separation at source in Danang city, Vietnam. The People's Committee of Danang city, Danang, Vietnam (in Vietnamese).

PCHC (2023) Thông báo số 188 /TB-UBND ngày 21 tháng 6 năm 2023 về Kết luận tại buổi họp nghe báo về Chương trình phân loại chất thải rắn sinh hoạt tại nguồn trên địa bàn thành phố Huế. The People's Committee of Hue City, Hue city, Vietnam.

Phuong NTT, Dajian Z, Phong LN (2015) Factors influencing waste separation intention of residential households in a developing country: Evidence from Hanoi, Vietnam. Habitat Int 48: 169-176. https://doi.org/10.1016/j.habitatint.2015.03.013

Ringle CM, Wende S, Becker J-M (2022) SmartPLS 4, 4. ed. Oststeinbek: SmartPLS GmbH.

SCNA (2021) Nghị quyết số 1264/NQ-UBTVQH14 ngày 27 tháng 4 năm 2021 về việc điều chỉnh địa giới hành chính các đơn vị hành chính cấp huyện và sắp xếp, thành lập các phường thuộc thành phố Huế, tỉnh Thừa Thiên Huế. Standing Committee of the National Assembly, Hanoi, Vietnam.

Tang D, Cai X, Nketiah E, Adjei M, Adu-Gyamfi G, Obuobi B (2023) Separate your waste: A comprehensive conceptual framework investigating residents' intention to adopt household waste separation. Sustain Prod Consum 39: 216-229. https://doi.org/10.1016/j.spc.2023.05.020

Thanh Hoa (2024) Huế - thành phố di sản và lễ hội. https://vietnam.vnanet.vn/vietnamese/long-form/hue-thanh-pho-di-san-va-le-hoi-306361.html#:~:text=Hu%E1%BA%BF%20hi%E1%BB%87n%20c%C3%B3%207%20di,)%3B%20v%C3%A0%202%20di%20s%E1%BA%A3n. (Accessed April 16 2024)

Wang S, Wang J, Yang S, Li J, Zhou K (2020) From intention to behavior: Comprehending residents' waste sorting intention and behavior formation process. Waste Manag 113: 41-50. https://doi.org/10.1016/j.wasman.2020.05.031

Wang S, Wang J, Zhao S, Yang S (2019) Information publicity and resident's waste separation behavior: An empirical study based on the norm activation model. Waste Manage 87: 33-42. https://doi.org/10.1016/j.wasman.2019.01.038

Yamane T (1973) Statistics: An Introductory Analysis, 3rd Edition ed. Harper and Row, New York, USA.

Zhang Y, Jing L, Bai Q, Shao W, Feng Y, Yin S, Zhang M (2018) Application of an integrated framework to examine Chinese consumers’ purchase intention toward genetically modified food. Food Qual Prefer 65: 118-128. https://doi.org/10.1016/j.foodqual.2017.11.001
